# Milk-derived osteopontin influences the composition of the intestinal intraepithelial lymphocyte compartment

**DOI:** 10.1093/immhor/vlaf057

**Published:** 2025-10-09

**Authors:** Kathleen G McClanahan, Jayden Capella, Jennifer A Gaddy, Danyvid Olivares-Villagómez

**Affiliations:** Department of Pathology, Microbiology, and Immunology, Vanderbilt University Medical Center, Nashville, TN, United States; Department of Pathology, Microbiology, and Immunology, Vanderbilt University Medical Center, Nashville, TN, United States; Department of Infectious Diseases, Vanderbilt University Medical Center, Nashville, TN, United States; Department of Infectious Diseases, Vanderbilt University Medical Center, Nashville, TN, United States

**Keywords:** IEL, intestines, milk, osteopontin

## Abstract

Osteopontin is a protein with many physiological roles widely expressed by many cell types, tissues, and bodily fluids, including breastmilk. The functions of breastmilk osteopontin are not clearly defined, however, it is known to impact intestinal and brain development in infants. Although it has been shown that endogenous osteopontin influences the survival of intestinal intraepithelial lymphocytes (IEL), the impact of milk osteopontin on developing intestinal immune cells remains unclear. In this report, mouse models lacking expression of osteopontin were used to demonstrate that milk-derived osteopontin is important for the development of IELs, with observed effects in both juvenile and adult mice. These changes are most prevalent in IELs expressing CD8αα: however, the impact of these alterations is unclear, as mice with disrupted IEL compartments are not more susceptible to intestinal inflammation induced by DSS or *Citrobacter rodentium* infection.

## Introduction

Infancy is a time of great physiological change and stress. This period is characterized by rapid growth and development, corresponding with exposure to numerous environmental factors. One of the main mechanisms through which the infant is exposed to the environment is through the gastrointestinal tract, which through its large surface area allows contact between the infant and numerous nutrients, microbes, and foreign factors. In early life, intestinal and immune development are not yet complete, resulting in a period of heightened susceptibility to pathogens and other stimuli. The main mechanism through which infants are protected during this period is breastfeeding. Breastmilk is a complex biological fluid composed of thousands of distinct compounds, the most prominent of which support infant nutrition, while others have bioactive potential. Bioactive proteins, including IgA, lactoferrin, and lysozyme play roles in protecting the infant from potential pathogens.^1–4^ Other components, including human milk oligosaccharides (HMOs), act as prebiotic factors, promoting the development of a robust intestinal microbiota and preventing the colonization of pathogenic organisms.^5^

One abundant bioactive protein present in breastmilk is osteopontin, a highly phosphorylated glycoprotein with numerous physiological roles. Originally isolated from rat bone matrix,^6^ osteopontin has been shown to impact calcification processes,^7^ cell migration,^8^^,^^9^ and immune cell function,^10^^,^^11^ among others. In addition to being found in most tissues, osteopontin is also present in many bodily fluids, including breastmilk.^12^

Mouse models have demonstrated the importance of milk-derived osteopontin in the development of the intestinal epithelium,^13^^,^^14^ as well as in brain myelination.^15^ Studies in rhesus macaques demonstrated that supplementation of formula with osteopontin led to an intestinal epithelial cell transcriptome more similar to that of breastfed infants.^16^ In human trials, infants fed osteopontin-supplemented formula showed a reduction in the inflammatory cytokine tumor necrosis factor (TNF)-α.^17^ In Europe, a bovine osteopontin supplement, known as Lacprodan^®^ OPN-10, has been approved for use in infant formula and has shown a robust safety profile.^18^^,^^19^ Given the potential for commercially available osteopontin-supplemented formulas in the near future, it is of critical importance to understand the effects of osteopontin on infant development.

One known target of osteopontin in the adult intestine is the intraepithelial lymphocyte (IEL) compartment. IEL are immune cells that reside between intestinal epithelial cells, interspersing at a ratio of ∼1 IEL for every 10 to 20 epithelial cells in the small intestine and ∼1 IEL for every 40 epithelial cells in the colon.^20^^,^^21^ IEL play critical roles in intestinal homeostasis, including immune tolerance and intestinal defense.^22–24^ There are different IEL subpopulations, many of which straddle the bridge between innate and adaptive immunity. The great majority of IEL express either TCR-αβ or TCR-γ; however, most IEL populations are limited in antigen recognition and/or respond to antigen through mechanisms other than canonical TCR signaling.^25^^,^^26^ The most prevalent IEL subsets include TCR-β^+^CD4^+^ and TCR-β^+^CD8-α^+^ IELs, as well as TCR-γ^+^ IELs in the small intestine. Of note, a large proportion of CD8α^+^ IEL do not express the canonical CD8-αβ co-receptor and instead express an alternate CD8-αα form. Because some IELs develop during early life,^27^ we hypothesize that milk-derived osteopontin may play a role in their development or maturation.

In this report, we found that osteopontin derived from maternal milk plays a role in IEL development, as mice that did not receive milk osteopontin displayed alterations in the IEL compartment both as juveniles (3 wk) and adults (8 wk). However, the impact of these alterations remains unclear, as mice with altered IEL compartments were not more susceptible to the tested models of intestinal inflammation.

## Materials and methods

### Mice

All experiments and animal procedures were conducted according to the NIH guidelines for the care and use of laboratory animals, under a protocol approved by the Institutional Animal Care and Use Committee at Vanderbilt University Medical Center. C57BL6/J and osteopontin KO (B6.129S6(Cg)-*Spp1^tm1Blh^*/J) mice were originally purchased from The Jackson Laboratory (000664 and 004936, respectively) and have been maintained in our colony for several years. Heterozygous mice were generated by crossing *Spp1*^−/−^ with WT C57BL6/J mice. F1 males (*Spp1*^+/−^) and females (*Spp1*^+/−^) were then crossed to generate *Spp1*^+/+^, *Spp1*^+/−^, and *Spp1*^−/−^ littermate offspring. Littermate *Spp1*^+/−^ and *Spp1*^−/−^ females were bred with *Spp1*^+/+^ males to generate pups for experiments. Females were harem bred (3 to 4 females and 1 male per cage) and maintained in the same breeding cage until external signs of pregnancy, at which point the pregnant female was removed to a fresh cage. Mice were maintained in a temperature (21°C to22°C) controlled room under a 12-hour light/dark cycle. Water and food were supplied *ad libitum*. Offspring were weaned at postnatal day (PND).^21^ No significant sex differences were found in experiments in this study, and male and female mice were used for all experiments except for those related to milk production.

### Cross-fostering of mouse pups

Littermate *Spp1*^+/−^ and *Spp1*^−/−^ dams were timed mated with *Spp1*^−/−^ sires. Briefly, *Spp1*^+/−^ and *Spp1*^−/−^ dams were maintained in exclusively female cages to allow synchronization of the estrous cycle. Female mice were then set to breed with singly housed *Spp1*^−/−^ sires that had been isolated for >5 d to allow for maximum sperm count. Females were left to breed for 1-4 days before being separated from the male. Females were observed for external signs of pregnancy, at which point they were separated into a clean cage. At PND0 or PND1, dams of time-matched litters were transferred between cages (*Spp1*^−/−^ dam to pups from *Spp1*^+/−^ dam and vice versa). Mice were monitored for signs of pup acceptance and feeding. Pups were left with the foster dam until use in an experiment or until weaning at PND21. If exact time-matched litters were not available, pups were occasionally switched on PND2 or PND3. In this case, the younger litter was always given to the *Spp1*^−/−^ dam to minimize milk osteopontin exposure.

### Milking of mouse dams

Dams were separated from litter into a clean cage 2 h prior to milking. After 2 h had elapsed, dams were injected i.p. with 2 IU of oxytocin (Sigma Aldrich, O3251) and placed back into the cage for 3 to 5 min while milk letdown was initiated. Dams were securely scruffed and milk was collected from at least 2 nipples using an adapted human breast pump (similar to that reported by^28^). Milk was stored at −70°C until analysis.

### Flush of mouse pup intestines

PND2, PND5, PND7, or PND21 pups were euthanized using CO_2_, followed by decapitation. The small intestines and colons (minus cecum) were removed and stored on ice in tubes containing 1 ml of phosphate-buffered saline (PBS) with cOmplete^TM^, EDTA-free Protease Inhibitor Cocktail (Roche, 04693132001, 1 tablet per 10 ml PBS). Intestines and their contents were homogenized using a handheld homogenizer (Fisherbrand) then centrifuged to pellet debris. Supernatant was transferred to clean tubes and stored at −70°C until analysis.

### Determination of osteopontin protein concentration

Previously frozen milk and intestinal flush samples were thawed at room temperature. Intestinal flush samples were centrifuged for 10 min at 10,000×*g*, 4 °C prior to analysis. Samples were analyzed using Osteopontin (OPN/SPP1) Mouse ELISA Kit (Invitrogen, EMSPP1), according to kit instructions. Intestinal samples were not diluted prior to analysis; milk samples were diluted 1:1,000,000 (1:1000 dilution followed by 1:1000 dilution).

### Lymphocyte isolation

Spleen and mesenteric lymph nodes (MLN) lymphocytes were isolated by conventional means.^29^ Briefly, cells were manually dispersed through a 70 mM cell strainer (ThermoFisher, 22-363-548) and ACK lysing buffer (KD Medical, RGF-3015) was applied to cell pellets to lyse red blood cells. Cells were recovered into cold RPMI (Gibco, 11835-030) supplemented with 10% fetal bovine serum (R&D Systems, S11150H), 2 mM L-glutamine (Corning, 25-005), 1x penicillin-streptomycin (Corning, 30-002), 10 mM HEPES (Corning, 25-060), and 25 mM 2-mercaptoethanol (Gibco, 21985-023). IEL cells were isolated by mechanical disruption as previously reported.^30^ Briefly, after flushing the intestinal contents with cold HBSS, cutting longitudinally, and removing excess mucus, the intestines were cut into small pieces (∼1 cm) and shaken for 45 min at 37 °C in HBSS supplemented with 5% fetal bovine serum and 2 mM EDTA (KD Medical, RGF-3130). Supernatants were recovered and cells isolated using a discontinuous 40%/70% Percoll (Cytiva, 17089101) gradient.

### Cell staining

Surface cell staining was performed following conventional techniques. For intracellular cytokine staining, cells were stimulated with Cell Activation Cocktail without Brefeldin A (BioLegend, 423302) in the presence of Golgi Stop (BD Biosciences, 51-2092KZ) for 4 h prior to staining. Extracellular markers were stained, cells were fixed briefly with 2% paraformaldehyde (Electron Microscopy Sciences, 15710-S), followed by permeabilization and intracellular staining using the BD Cytofix/Cytoperm kit (BD Biosciences, 554714) according to manufacturer’s instructions. All stained samples were acquired using BD FACS Canto II or BD LSRFortessa (BD Biosciences). Data were analyzed using FlowJo software (FlowJo LLC). Antibodies used can be found in Table 1. Gating strategies can be found in Fig. S1.

**Table 1.** Antibodies used for extracellular and intracellular staining.

Antibody	Clone	Supplier	Catalogue No.	Dilution
Annexin V APC	…	Cytek Biosciences	20-6409	1:100
CD107a APC-Cy7	1D4B	BioLegend	121615	1:200
CD19 Biotin	1D3	Cytek Biosciences	30-0193	1:200
CD314 PE-Dazzle 594	CX5	BioLegend	130213	1:200
CD4 FITC	GK1.5	Cytek Biosciences	35-0041	1:200
CD45 PE-Cy7	30-F11	BD Biosciences	561868	1:300
CD8α APC-H7	53-6.7	BD Biosciences	560247	1:200
CD8α violetFluor 500	53-6.7	Cytek Biosciences	85-0081	1:200
CD8β PE	YTS156.7.7	BioLegend	126607	1:1000
Ghost Dye^TM^ UV 450	–	Cytek Biosciences	13-0868	1:1000
Ghost Dye^TM^ Violet 510	–	Cytek Biosciences	13-0870	1:1000
GzB APC	NGZB	Invitrogen	17-8898	1:200
IFN-γ PerCP-Cy5-5	XMG1.2	BioLegend	505821	1:200
Ki67 APC	16A8	BioLegend	652405	1:200
Streptavidin BV421	–	BioLegend	405226	1:200
TCR-β Alexa Fluor 700	H57-597	BioLegend	109223	1:200
TCR-β PerCP-Cy5-5	H57-597	Cytek Biosciences	65-5961	1:200
TCR-γ eFluor450	GL3	Invitrogen	48-5711	1:200

### DSS-induced colitis

Mice were supplied with 3% DSS (Thermo Scientific, J63606.22) in the drinking water for 5 d before being switched back to sterile water. Mice were weighed daily and monitored for signs of disease, including rectal inflammation, diarrhea, and wasting. For the recovery model, mice were exposed to 4% DSS for 5 d before returning to sterile water.

### *Citrobacter rodentium* infection

Mice were infected with ∼1 × 10^9^ CFU of *Citrobacter rodentium* (ATCC, 51459) by oral gavage. Mice were weighed daily and monitored for signs of disease, including rectal inflammation, diarrhea, and wasting, until harvest at 14 d post-infection.

### Statistical analysis

Data were analyzed using Prism 9 (GraphPad, San Diego, California, USA) to determine significant (*P* < 0.05) differences between groups. Comparisons between 2 groups were made by Student *t* test. Comparisons between more than 2 groups were made by 1-way ANOVA with post hoc Tukey HSD Test. Data are presented as mean ± standard error of the mean.

## Results

**Figure 1.**
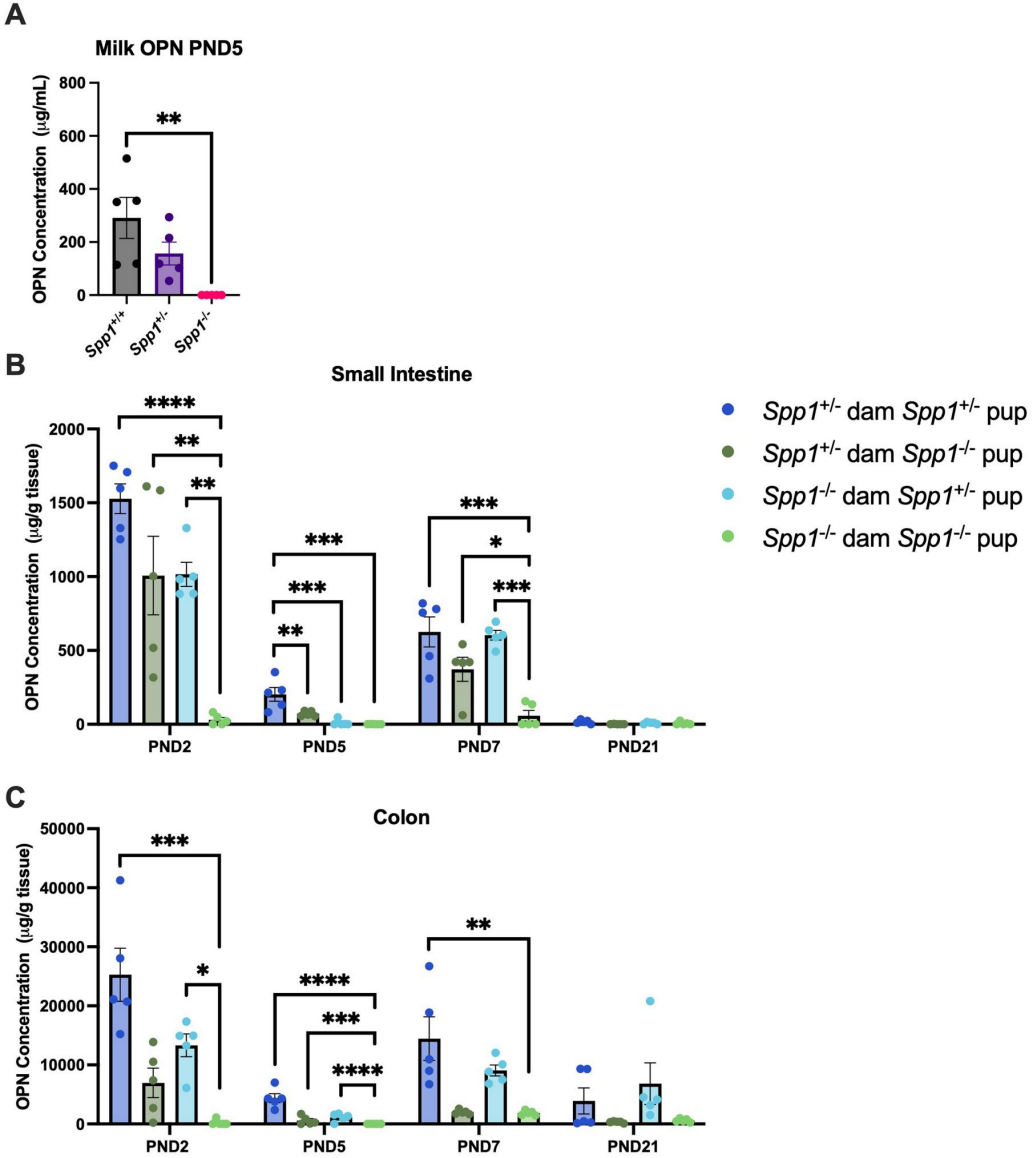
Osteopontin protein expression in milk and intestines of *Spp1*^+/+^, *Spp1*^+/−^ and *Spp1*^−/−^ mice. (A) Osteopontin concentration in postpartum day 5 milk from the indicated dams. (B) Osteopontin concentration in juvenile small intestinal homogenate. (C) Osteopontin concentration in juvenile colonic homogenate. Each symbol represents an individual mouse, *n* = 5. Bars indicate SEM. Data are from 2 independent experiments. *P ≤ 0.05; ** *P* ≤ 0.01; ***P ≤ 0.001; ****P ≤ 0.0001.

To determine osteopontin milk concentration in our mouse models, we analyzed milk from *Spp1*^+/+^, *Spp1*^+/−^, and *Spp1*^−/−^ dams from our colony at 5 d post-partum (PND5) (Fig. 1A). As expected, milk from *Spp1*^−/−^ dams had no detectible osteopontin. It is well-known that milk osteopontin concentration varies widely between individuals,^15^^,^^31^^,^^32^ which was reflected in our samples. On average, *Spp1*^+/−^ dams had roughly half the osteopontin concentration of *Spp1*^+/+^ dams; however, inter-group variance was large, with *Spp1*^+/+^ mice having milk osteopontin concentrations between 100 and 500 µg/ml, and *Spp1*^+/−^ mice having concentrations between 50 and 300 µg/ml. It is important to note that estimations in osteopontin concentration in Fig. 1A were obtained from dams at diverse ages, rearing litters of different sizes, which may impact the concentration of osteopontin in milk. Despite these caveats, *Spp1*^+/−^ dams showed reduced concentration levels as expected for heterozygosity.

We then investigated the effects of maternal and pup genotype on intestinal osteopontin by analyzing small intestinal and colon contents of *Spp1*^+/−^ and *Spp1*^−/−^ pups from *Spp1*^+/−^ (osteopontin-competent) dams crossed with *Spp1*^−/−^ sires, and *Spp1*^−/−^ (osteopontin-deficient) dams crossed with *Spp1*^+/−^ sires. We observed that osteopontin concentration in the small intestine of *Spp1*^+/−^ pups reared by osteopontin competent dams (dark blue bars) peaks early in life at approximately 1500 µg OPN/g tissue on PND2, followed by a decrease in osteopontin concentration to roughly 250 µg/g at PND5 that rebounds to approximately 600 µg/g by PND7. By PND21, little to no osteopontin is detectible (Fig. 1B). This pattern was also observed in the colon, though at concentrations roughly 10× of that seen in the small intestine (Fig. 1C). In similar fashion, *Spp1*^−/−^ pups reared by *Spp1*^+/−^ dams, and *Spp1*^+/−^ pups reared by *Spp1*^−/−^ dams also show a fluctuating patter in intestinal osteopontin concentration (Fig. 1B dark green and light blue bars respectively). In the former, the observed osteopontin in the intestines is fully derived from the mother’s milk; however, in the latter group, the source of intestinal osteopontin is the pups. It is important to note that due to the impracticality of collecting intestinal flushes from young pups, the intestines were homogenized; thus, what it is observed in *Spp1*^+/−^ pups reared by *Spp1*^−/−^ dams may represent osteopontin not secreted into the intestinal lumen, but instead protein present in other intestinal sites. The fluctuating pattern was unexpected, as osteopontin concentration in mouse milk has been shown to peak at 1 d after birth before falling off quickly and reaching lower levels by day 5.^15^ As this fluctuating pattern was observed even in pups only receiving milk osteopontin, *Spp1*^−/−^ pups reared by *Spp1*^+/−^ dams (dark green bars), the rebound of intestinal osteopontin concentrations at day 7 could potentially be explained by fluctuations in milk osteopontin concentration. However, pup intake of milk increases over time, as does the total volume of the pup intestinal lumen. Thus, the fluctuating pattern may more accurately represent an increase in gross osteopontin intake or intestinal capacity during this time.

As expected, no osteopontin was seen in the intestines of *Spp1*^−/−^ pups from *Spp1*^−/−^ dams at any time point (Fig. 1B, light green bars). Of note, osteopontin was readily detectable in the intestines of *Spp1*^−/−^ pups reared by *Spp1*^+/−^ dams, indicating the presence of milk derived osteopontin in the lumen of these mice (Fig. 1B, dark green bars). Comparing the amount of osteopontin present in the intestines of these mice (dark green bars) to the total present in *Spp1*^+/−^ pups reared by *Spp1*^+/−^ dams (dark blue bars) yields an estimate of milk osteopontin contribution to the total osteopontin detected in the intestine. In the small intestine, the milk contribution starts at around 66% on day 2, decreasing to 36% on day 5, before increasing to 60% on day 7. At day 21, the milk contribution is negligible, at 6%. In the colon, the milk contribution starts at 27%, falling to 13% on day 5 and 14% on day 7, ending at 8% on day 21. As mentioned above, osteopontin concentration measured in *Spp1*^+/−^ pups has the potential caveat of detecting non-luminal intestinal osteopontin.

**Figure 2.**
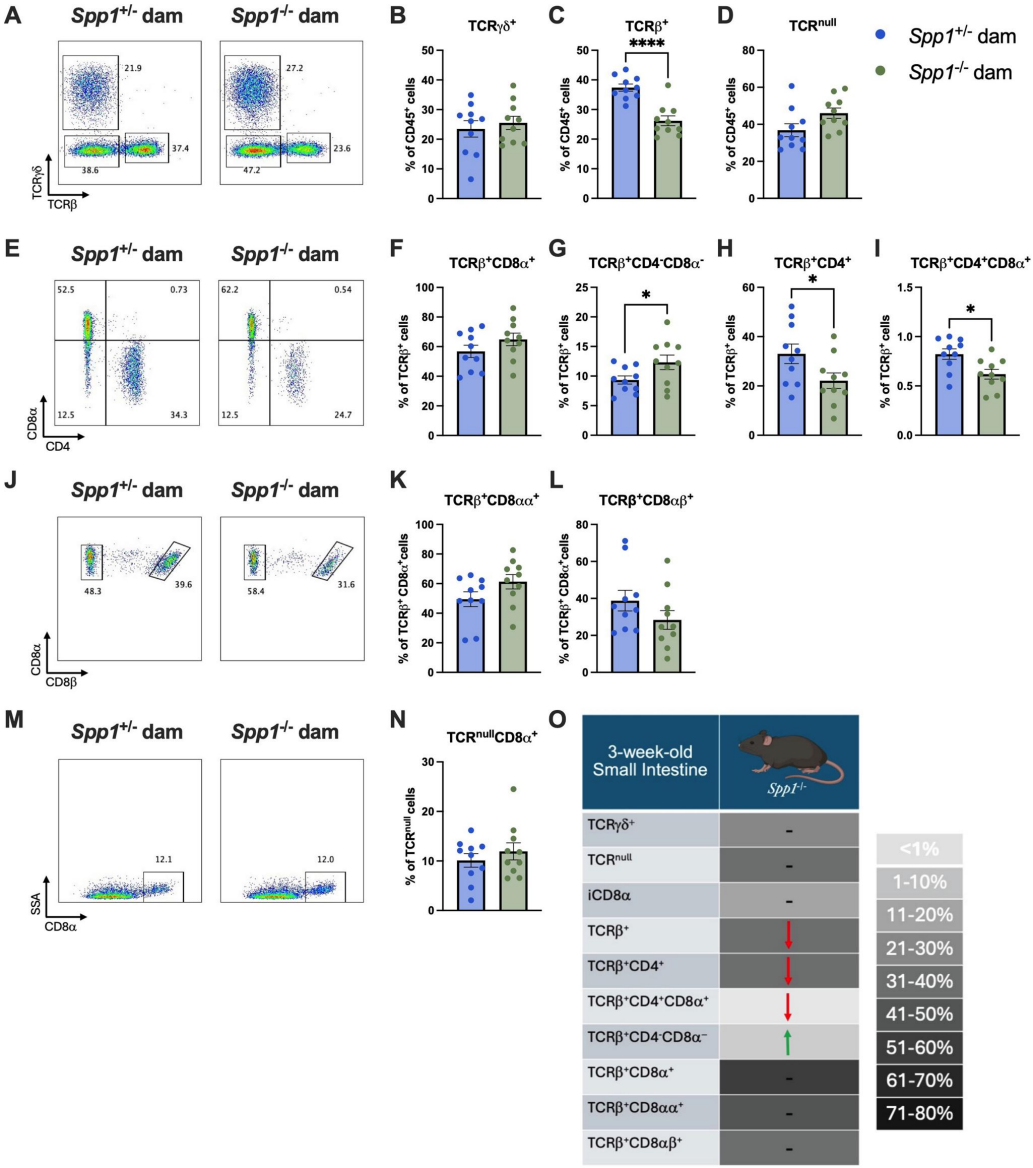
IEL frequencies in the small intestine of PND21 *Spp1*^+/−^ mice birthed and reared by *Spp1*^+/−^ and *Spp1*^−/−^ dams. (A) Representative flow plot of live CD45^+^ population gated by expression of TCR-β x-axis) and TCR-γ (*y*-axis). (B) Quantification of TCR-γ^+^ cells. (C) Quantification of TCR-β^+^ cells. (D) Quantification of TCR^null^ cells. (E) Representative flow plot of CD45^+^TCR-β^+^ population gated by expression of CD4 *x*-axis) and CD8-α (*y*-axis). (F) Quantification of TCR-β^+^CD8-α^+^ cells. (G) Quantification of TCR-β^+^CD4^-^CD8-α^-^ cells. (H) Quantification of TCR-β^+^CD4^+^ cells. (I) Quantification of TCR-β^+^CD4^+^CD8α^+^ cells. (J) Representative flow plot of CD45^+^TCR-β^+^CD8-α^+^ population gated by expression of CD8- *x*-axis). (K) Quantification of TCR-β^+^CD8-αα^+^ cells. (L) Quantification of TCR-β^+^CD8-αβ^+^ cells. (M) Representative flow plot of CD45^+^TCR^null^ population gated by expression of CD8-α *x*-axis). (N) Quantification of TCR^null^CD8-α^+^ cells. (O) Summary of changes in 3-wk-old small intestinal IEL. The prevalence of given IEL populations in mice raised by osteopontin-competent (*Spp1*^+/−^) dams is depicted by shading of the indicated cell (darker color equivalent to higher prevalence, key shown to right of table). Arrows within cells indicate an increase or decrease in prevalence of the given IEL population in pups raised by osteopontin-deficient (*Spp1*^−/−^) dams as compared to pups from osteopontin-competent (*Spp1*^+/−^) dams. Dashes indicate no significant difference in the given population. Each dot represents an individual mouse, *n* = 10. Bars indicate SEM. Data are from 2 independent experiments. *P ≤ 0.05; ****P ≤ 0.0001.

We then sought to determine the effects of milk-derived osteopontin on the development of the IEL compartment. Thus, we generated *Spp1*^+/−^ pups from littermate *Spp1*^+/−^ and *Spp1*^−/−^ dams crossed with *Spp1*^+/+^ sires and analyzed their IEL compartment at 3 and 8 wk of life. Due to the great variation within samples in total IEL cellularity, most data are portrayed as cellular frequencies. At 3 wk (Fig. 2A–N), no differences were observed in TCR-γ^+^ and TCR^null^ IEL (Fig. 2A, B, and D). However, a significant reduction in frequency of total TCR-β ^+^ IEL were observed in pups reared by osteopontin-deficient dams (Fig. 2C). Within the TCRβ^+^ fraction, pups from *Spp1*^−/−^ dams had an increased proportion of CD4^-^CD8-α^-^ IEL and a decreased proportion of CD4^+^ and CD4^+^CD8-α ^+^ IEL (Fig. 2E, G–I). The remaining IEL populations showed no differences (Fig. 2F, J–N). A summary of these results is presented in Fig. 2O.

**Figure 3.**
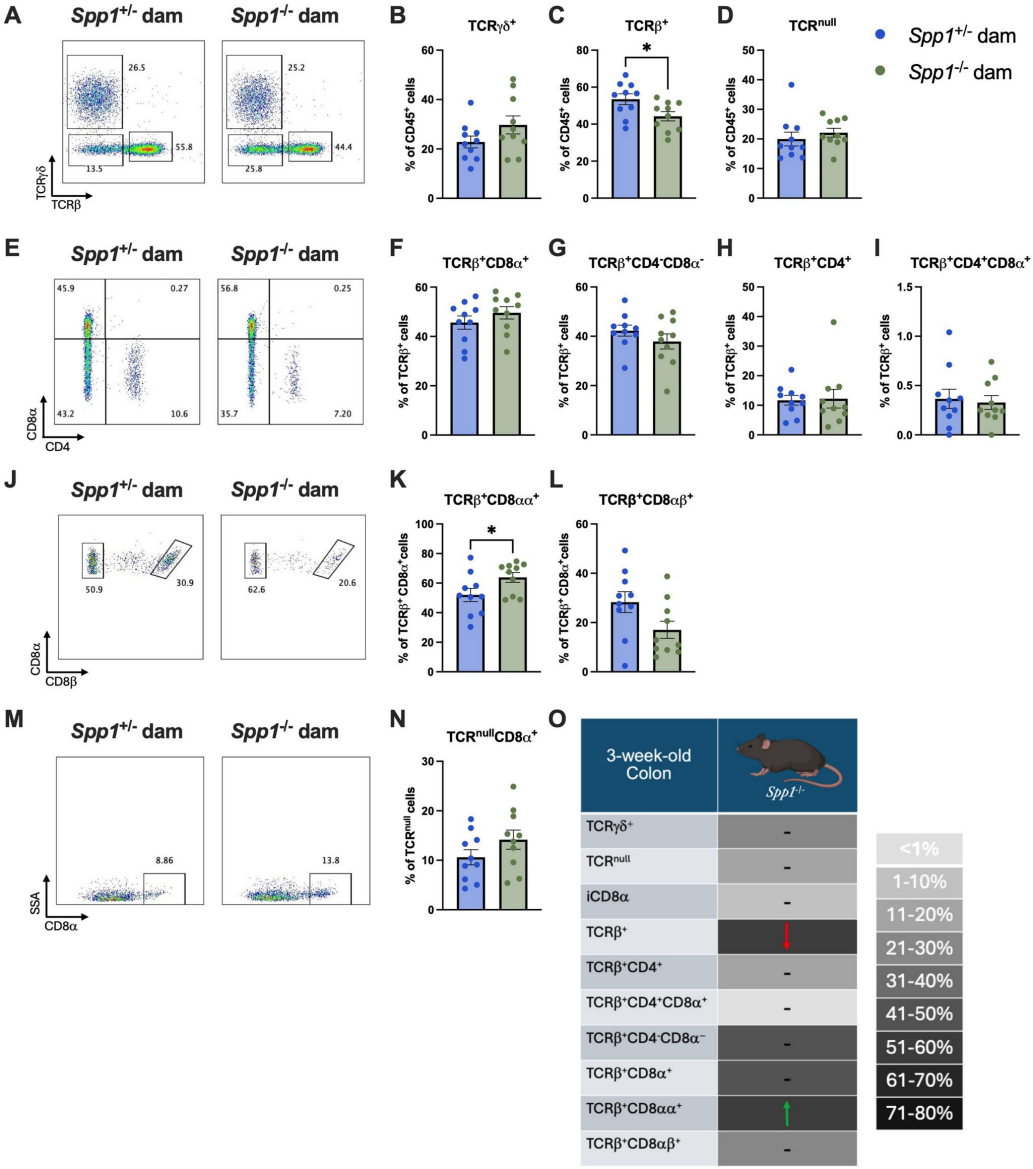
IEL frequencies in the colon of PND21 *Spp1*^+/−^ mice birthed and reared by *Spp1*^+/−^ and *Spp1*^−/−^ dams. (A) Representative flow plot of live CD45^+^ population gated by expression of TCR--β *x*-axis) and TCR-γ (*y*-axis). (B) Quantification of TCR-γ^+^ cells. (C) Quantification of TCR-β^+^ cells. (D) Quantification of TCR^null^ cells. (E) Representative flow plot of CD45^+^TCR-β^+^ population gated by expression of CD4 *x*-axis) and CD8-α (*y*-axis). (F) Quantification of TCR-β^+^CD8-α^+^ cells. (G) Quantification of TCR-β^+^CD4^-^CD8-α^−^ cells. (H) Quantification of TCR-β^+^CD4^+^ cells. (I) Quantification of TCR-β^+^CD4^+^CD8-α^+^ cells. (J) Representative flow plot of CD45^+^TCR-β^+^CD8-α^+^ population gated by expression of CD8-β*x*-axis). (K) Quantification of TCR-β^+^CD8-αα^+^ cells. (L) Quantification of TCR-β^+^CD8αβ^+^ cells. (M) Representative flow plot of CD45^+^TCR^null^ population gated by expression of CD8-α*x*-axis). (N) Quantification of TCR^null^CD8-α^+^ cells. (O) Summary of changes in 3-wk-old small intestinal IEL. The prevalence of given IEL populations in mice raised by osteopontin-competent (*Spp1*^+/−^) dams is depicted by shading of the indicated cell (darker color equivalent to higher prevalence, key shown to right of table). Arrows within cells indicate an increase or decrease in prevalence of the given IEL population in pups raised by osteopontin-deficient (*Spp1*^−/−^) dams as compared to pups from osteopontin-competent (*Spp1*^+/−^) dams. Dashes indicate no significant difference in the given population. Each dot represents an individual mouse, *n* = 10. Bars indicate SEM. Data are from 2 independent experiments. *P ≤ 0.05.

In terms of the colon (Fig. 3A–N), a decrease in TCR-β^+^ IEL was also observed (Fig. 3C), and within this population, pups from *Spp1*^−/−^ dams had an increase in CD8-αα^+^ IEL (Fig. 3K). The remaining IEL populations were similar between groups (Fig. 3A, B, D, E–N). A summary of these results is presented in Fig. 3O.

**Figure 4.**
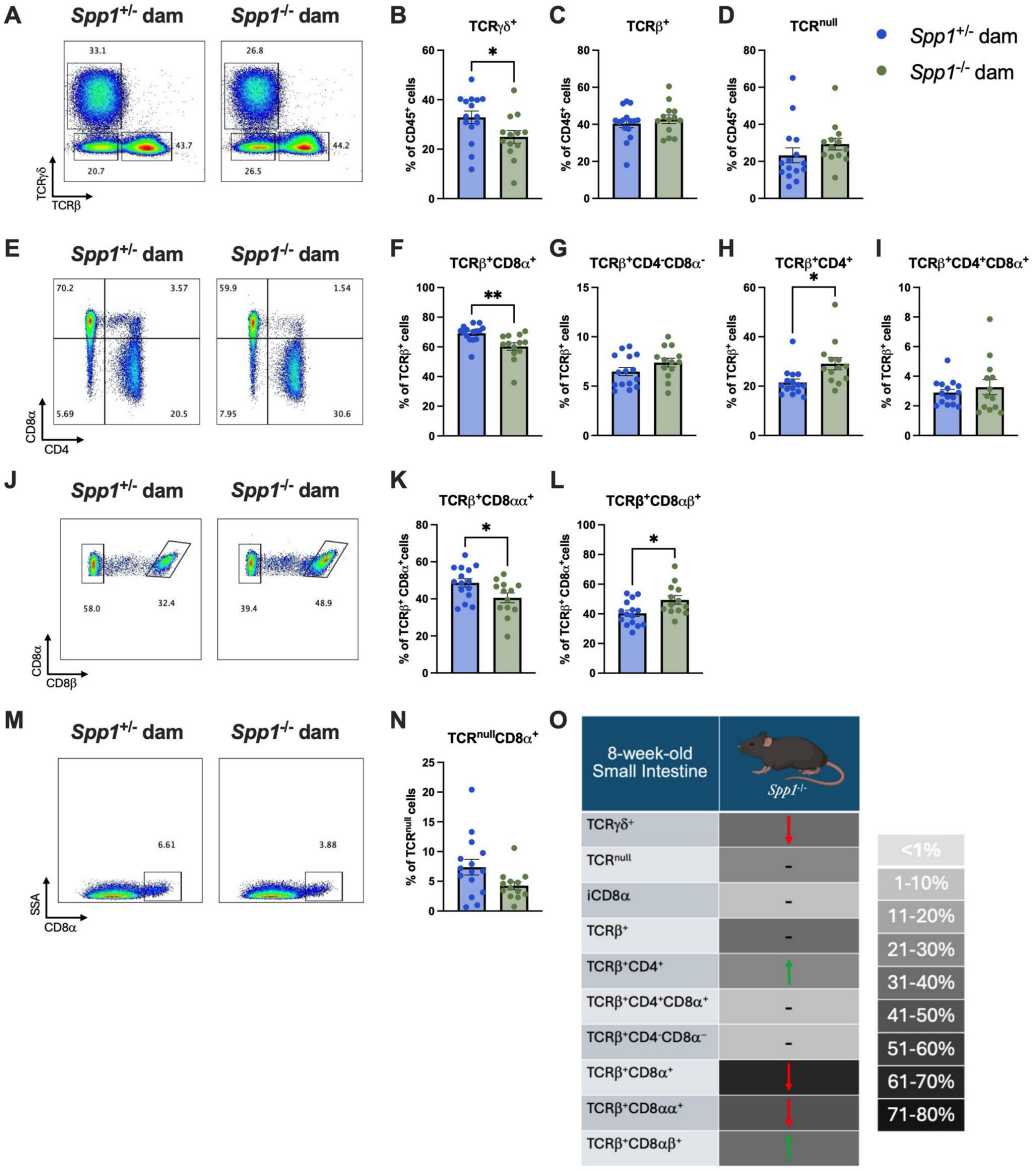
IEL frequencies in the small intestine of 8-week-old *Spp1*^+/−^ mice birthed and reared by *Spp1*^+/−^ and *Spp1*^−/−^ dams. (A) Representative flow plot of live CD45^+^ population gated by expression of TCR-*x*-axis) and TCR-γ (*y*-axis). (B) Quantification of TCR-γ^+^ cells. (C) Quantification of TCR-β^+^ cells. (D) Quantification of TCR^null^ cells. (E) Representative flow plot of CD45^+^TCR-β^+^ population gated by expression of CD4 x-axis) and CD8-α (*y*-axis). (F) Quantification of TCR-β^+^CD8-α^+^ cells. (G) Quantification of TCR-β^+^CD4^-^CD8-α^−^ cells. (H) Quantification of TCR-β^+^CD4^+^ cells. (I) Quantification of TCRβ^+^CD4^+^CD8α^+^ cells. (J) Representative flow plot of CD45^+^TCR-β^+^CD8-α^+^ population gated by expression of CD8β*x*-axis). (K) Quantification of TCRβ^+^CD8-αα^+^ cells. (L) Quantification of TCRβ^+^CD8-αβ^+^ cells. (M) Representative flow plot of CD45^+^TCR^null^ population gated by expression of CD8-*x*-axis). (N) Quantification of TCR^null^CD8α^+^ cells. (O) Summary of changes in 3-week-old small intestinal IEL. The prevalence of given IEL populations in mice raised by osteopontin-competent (*Spp1*^+/−^) dams is depicted by shading of the indicated cell (darker color equivalent to higher prevalence, key shown to right of table). Arrows within cells indicate an increase or decrease in prevalence of the given IEL population in pups raised by osteopontin-deficient (*Spp1*^−/−^) dams as compared to pups from osteopontin-competent (*Spp1*^+/−^) dams. Dashes indicate no significant difference in the given population. Each dot represents an individual mouse, *n* = 15 (*Spp1*^+/−^ dam), *n* = 13 (*Spp1*^−/−^ dam). Bars indicate SEM. Data are from two independent experiments. *P ≤ 0.05, **P ≤ 0.01.

At 8 wk (Fig. 4A–N), pups from *Spp1*^−/−^ dams had a decreased proportion of TCR- γ^+^ IEL (Fig. 4B) in the small intestine. While the proportion of total TCR-β^+^ IEL was not different between groups (Fig. 4C), pups from *Spp1*^−/−^ dams had decreased frequency in TCRβ^+^CD8-α ^+^ IEL and a corresponding increase in TCR-β^+^CD4^+^ IEL (Fig. 4F, H). Within the TCR-β^+^CD8-α^+^ fraction, there was an observed decrease in CD8-αα^+^ IEL and a corresponding increase in CD8-αβ^+^ IEL (Fig. 4J–L). The remaining IEL populations were unchanged (Fig. 4A, D, E, G, I, M, N). A summary of these results in presented in Fig. 4O.

**Figure 5.**
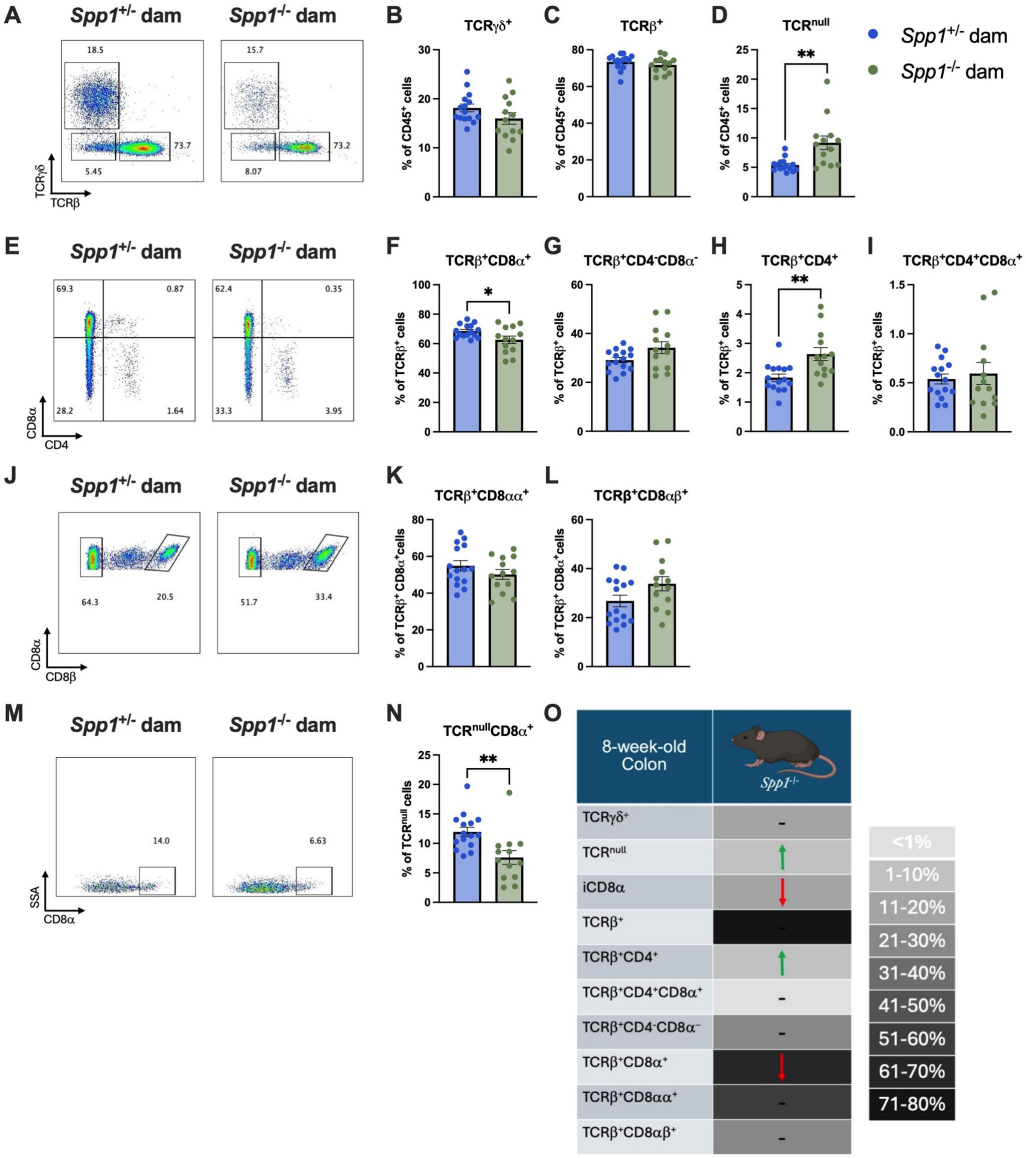
IEL frequencies in the colon of 8-wk-old *Spp1*^+/−^ mice birthed and reared by *Spp1*^+/−^ and *Spp1*^−/−^ dams. (A) Representative flow plot of live CD45^+^ population gated by expression of TCR-β *x*-axis) and TCR-γ (*y*-axis). (B) Quantification of TCR-γ^+^ cells. (C) Quantification of TCR-β^+^ cells. (D) Quantification of TCR^null^ cells. (E) Representative flow plot of CD45^+^TCR-β^+^ population gated by expression of CD4 *x*-axis) and CD8α (*y*-axis). (F) Quantification of TCR-β^+^CD8-α^+^ cells. (G) Quantification of TCR-β^+^CD4^-^CD8-α^−^ cells. (H) Quantification of TCR-β^+^CD4^+^ cells. (I) Quantification of TCR-β^+^CD4^+^CD8α^+^ cells. (J) Representative flow plot of CD45^+^TCRβ^+^CD8α^+^ population gated by expression of CD8-β *x*-axis). (K) Quantification of TCR-β^+^CD8-αα^+^ cells. (L) Quantification of TCR-β^+^CD8-αβ^+^ cells. (M) Representative flow plot of CD45^+^TCR^null^ population gated by expression of CD8-α*x*-axis). (N) Quantification of TCR^null^CD8-α^+^ cells. (O) Summary of changes in 3-wk-old small intestinal IEL. The prevalence of given IEL populations in mice raised by osteopontin-competent (*Spp1*^+/−^) dams is depicted by shading of the indicated cell (darker color equivalent to higher prevalence, key shown to right of table). Arrows within cells indicate an increase or decrease in prevalence of the given IEL population in pups raised by osteopontin-deficient (*Spp1*^−/−^) dams as compared to pups from osteopontin-competent (*Spp1*^+/−^) dams. Dashes indicate no significant difference in the given population. Each dot represents an individual mouse; *n* = 15 (*Spp1*^+/−^ dam), *n* = 13 (*Spp1*^−/−^ dam). Bars indicate SEM. Data are from two independent experiments. *P ≤ 0.05, **P ≤ 0.01.

In the colon (Fig. 5A–N), pups from *Spp1*^−/−^ dams had an increase in TCR^null^ IEL and decreased frequencies inTCR^null^CD8-α^+^ IEL (Fig. 5D, M, N). Within the TCR-β^+^ fraction, there was a decrease in TCR-β^+^CD8-α^+^ IEL and a corresponding increase in TCR-β^+^CD4^+^ IEL (Fig. 5F, H), similar to that seen in the small intestine. The remaining populations did not fluctuate between groups (Fig. 5A–C, E, G, I, J–L). A summary of these results in presented in Fig. 5O.

In summary, the IEL population frequencies in the small intestine and colon of pups derived from *Spp1*^+/−^ dams differed from pups reared by *Spp1*^−/−^ dams (Figs 2O, 3O, 4O, 5O). The differences observed varied between recently weaned and adult mice, and also within specific IEL populations.

**Figure 6.**
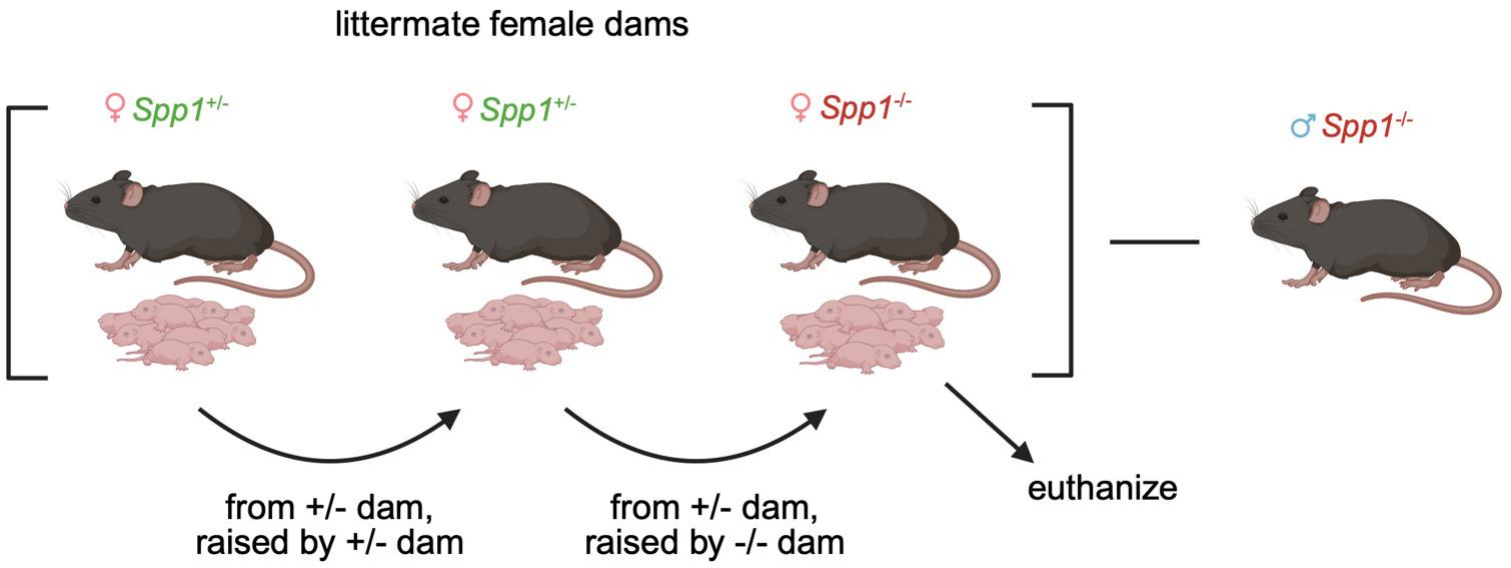
Schematic of cross-foster cohort design. Age-matched litters from timed-mated *Spp1*^+/−^ and *Spp1*^−/−^ littermate dams mated with *Spp1*^−/−^ sires were switched as follows: litter from *Spp1*^+/−^ dam to second *Spp1*^+/−^ dam, litter from second *Spp1*^+/−^ dam to *Spp1*^−/−^ dam. The litter from the *Spp1*^−/−^ dam was euthanized and not used in this experiment.

As this model was limited in pup genotype (only *Spp1*^+/−^ pups were analyzed) and only partially controlled for maternal microbiota effects, we generated a cross-fostered model in which littermate *Spp1*^+/−^ and *Spp1*^−/−^ dams were timed mated with *Spp1*^−/−^ sires. Upon birth of 3 synchronized litters (2 from *Spp1*^+/−^ dams and 1 from *Spp1*^−/−^ dam), dams were transferred between cages to foster each other’s pups (pups from *Spp1*^+/−^ dam to other *Spp1*^+/−^ dam, pups from *Spp1*^+/−^ dam to *Spp1*^−/−^ dam, pups from *Spp1*^−/−^ dam not used for this experiment [Fig. 6]). This model controls for vertical transfer of microbiota during birth, as all pups analyzed in these experiments come from *Spp1*^+/−^ dams. Furthermore, this strategy generated both *Spp1*^+/−^ and *Spp1*^−/−^ pups, allowing for analysis of both maternal and pup contributions to intestinal osteopontin.

**Figure 7.**
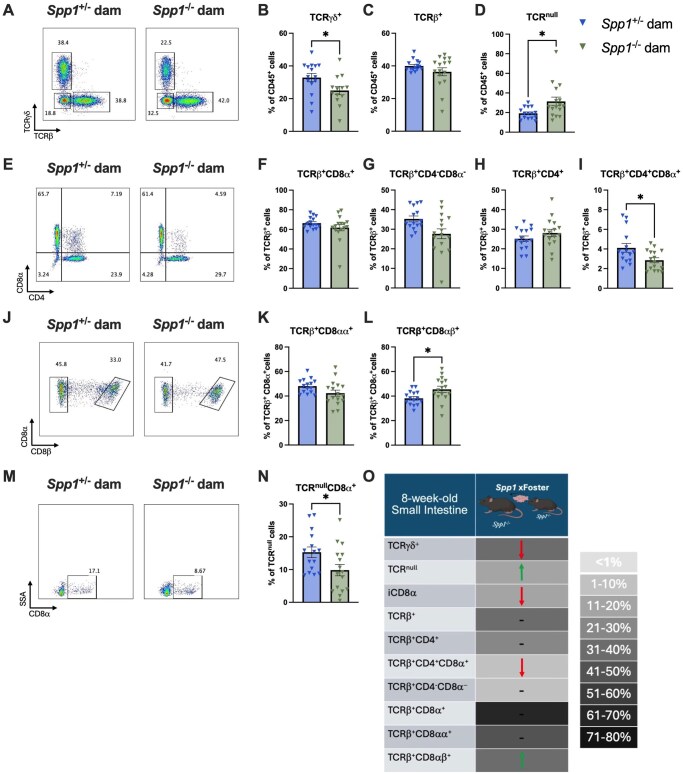
IEL frequencies in the small intestine of 8-week-old cross-fostered mice. (A) Representative flow plot of live CD45^+^ population gated by expression of TCR[-β (*x*-axis) and TCR-γ (*y*-axis). (B) Quantification of TCR-γ^+^ cells. (C) Quantification of TCRβ^+^ cells. (D) Quantification of TCR^null^ cells. (E) Representative flow plot of CD45^+^TCR-β^+^ population gated by expression of CD4 (x-axis) and CD8α (y-axis). (F) Quantification of TCRβ^+^CD8-α^+^ cells. (G) Quantification of TCRβ^+^CD4^-^CD8α^−^ cells. (H) Quantification of TCR-β^+^CD4^+^ cells. (I) Quantification of TCRβ^+^CD4^+^CD8α^+^ cells. (J) Representative flow plot of CD45^+^TCRβ^+^CD8α^+^ population gated by expression of CD8-β (*x*-axis). (K) Quantification of TCRβ^+^CD8αα^+^ cells. (L) Quantification of TCR-β^+^CD8-αβ^+^ cells. (M) Representative flow plot of CD45^+^TCR^null^ population gated by expression of CD8α (*x*-axis). (N) Quantification of TCR^null^CD8α^+^ cells. (O) Summary of changes in 3-k-old small intestinal IEL. The prevalence of given IEL populations in mice raised by osteopontin-competent (*Spp1*^+/−^) dams is depicted by shading of the indicated cell (darker color equivalent to higher prevalence, key shown to right of table). Arrows within cells indicate an increase or decrease in prevalence of the given IEL population in pups raised by osteopontin-deficient (*Spp1*^−/−^) dams as compared to pups from osteopontin-competent (*Spp1*^+/−^) dams. Dashes indicate no significant difference in the given population. Each dot represents an individual mouse; *n* = 15 (*Spp1*^+/−^ dam), *n* = 16 (*Spp1*^−/−^ dam). Bars indicate SEM. Data are from two independent experiments. *P ≤ 0.05.

To determine the long-term effect of fostering with osteopontin-competent and -deficient dams, we analyzed the IEL compartment at 8w of age. At 8w (Fig. 7A–N), mice fostered by osteopontin-deficient dams had a higher percentage of TCR^null^ IEL (Fig. 7D), along with a lower percentage of TCR-γ^+^ and TCR^null^CD8-α^+^ IEL (Fig. 7B, M, N). Within the TCR-β^+^ subset, mice fostered by osteopontin-deficient dams had a decreased percentage of CD4^+^CD8-α^+^ IEL (Fig. 7I) and an increased percentage of CD8-αβ^+^ IEL (Fig. 7L). Other IEL populations were not affected (Fig. 7A, C, E–H, J, K). A summary of these results in presented in Fig. 7O.

**Figure 8.**
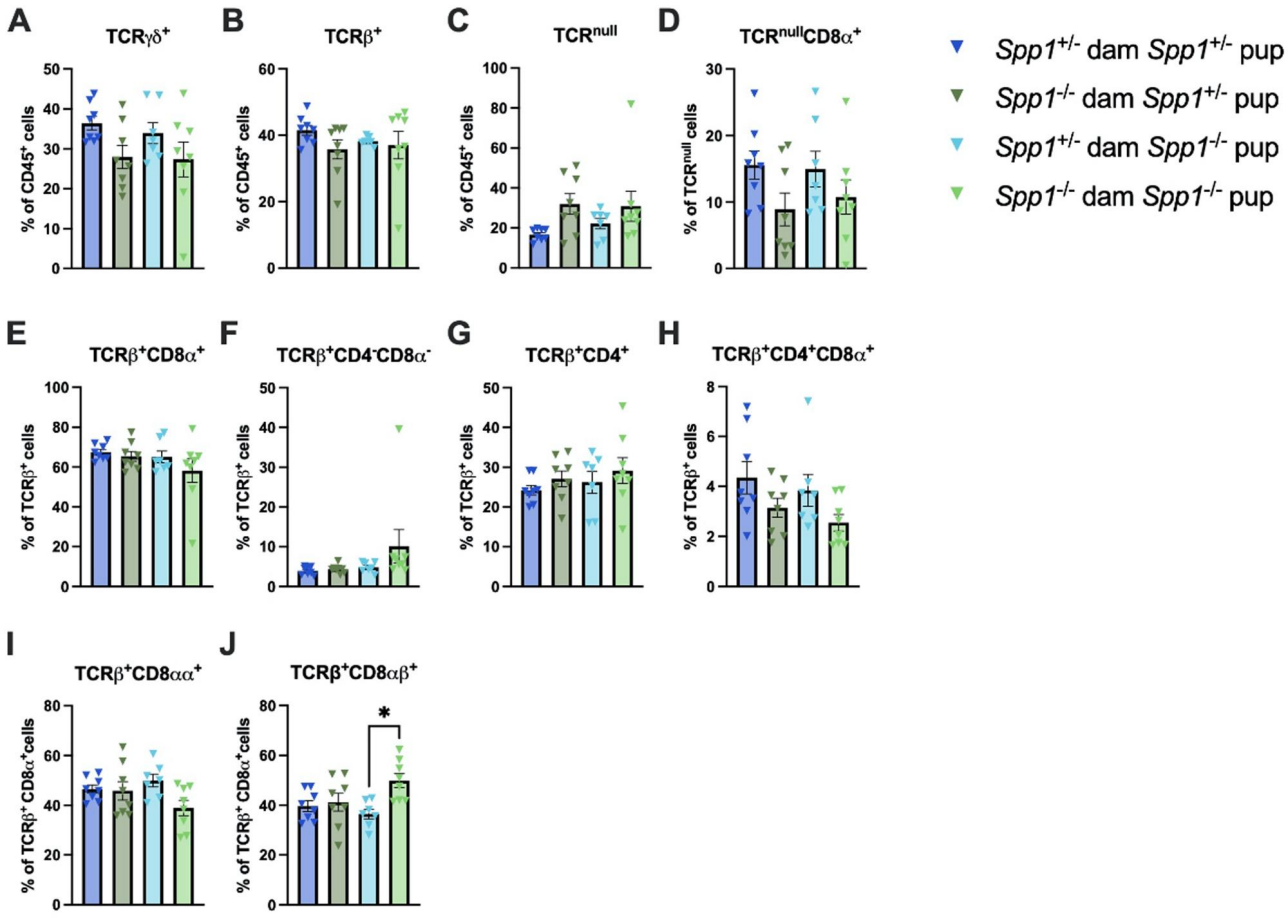
IEL frequencies in the small intestine of 8-week-old cross-fostered *Spp1*^+/−^ and *Spp1*^−/−^ mice. (A) Quantification of TCR-γ^+^ cells. (B) Quantification of TCR-β^+^ cells. (C) Quantification of TCR^null^ cells. (D) Quantification of TCR^null^CD8α^+^ cells. (E) Quantification of TCR[-β^+^CD8-α^+^ cells. (F) Quantification of TCRβ^+^CD4^-^CD8α^−^ cells. (G) Quantification of TCR-β^+^CD4^+^ cells. (H) Quantification of TCR-β^+^CD4^+^CD8-α^+^ cells. (I) Quantification of TCR-β^+^CD8-αα^+^ cells. (J) Quantification of TCR-β^+^CD8-αβ^+^ cells. Each dot represents an individual mouse; n = 8 (*Spp1*^+/−^ dam *Spp1*^+/−^ pup), *n* = 8 (*Spp1*^−/−^ dam *Spp1*^+/−^ pup), *n* = 7 (*Spp1*^+/−^ dam *Spp1*^−/−^ pup), *n* = 8 (*Spp1*^−/−^ dam *Spp1*^−/−^ pup). Bars indicate SEM. Data are from 2 independent experiments. *P ≤ 0.05, **P ≤ 0.01.

When divided by both dam and pup genotype (Fig. 8A–J), fewer differences were noted, with only a slight increase in TCR-β^+^CD8-αβ^+^ IEL in *Spp1*^−/−^ pups from *Spp1*^−/−^ dams as compared to *Spp1*^−/−^ pups from *Spp1*^+/−^ (Fig. 8J) and no other noted differences (Fig. 8A–I).

**Figure 9.**
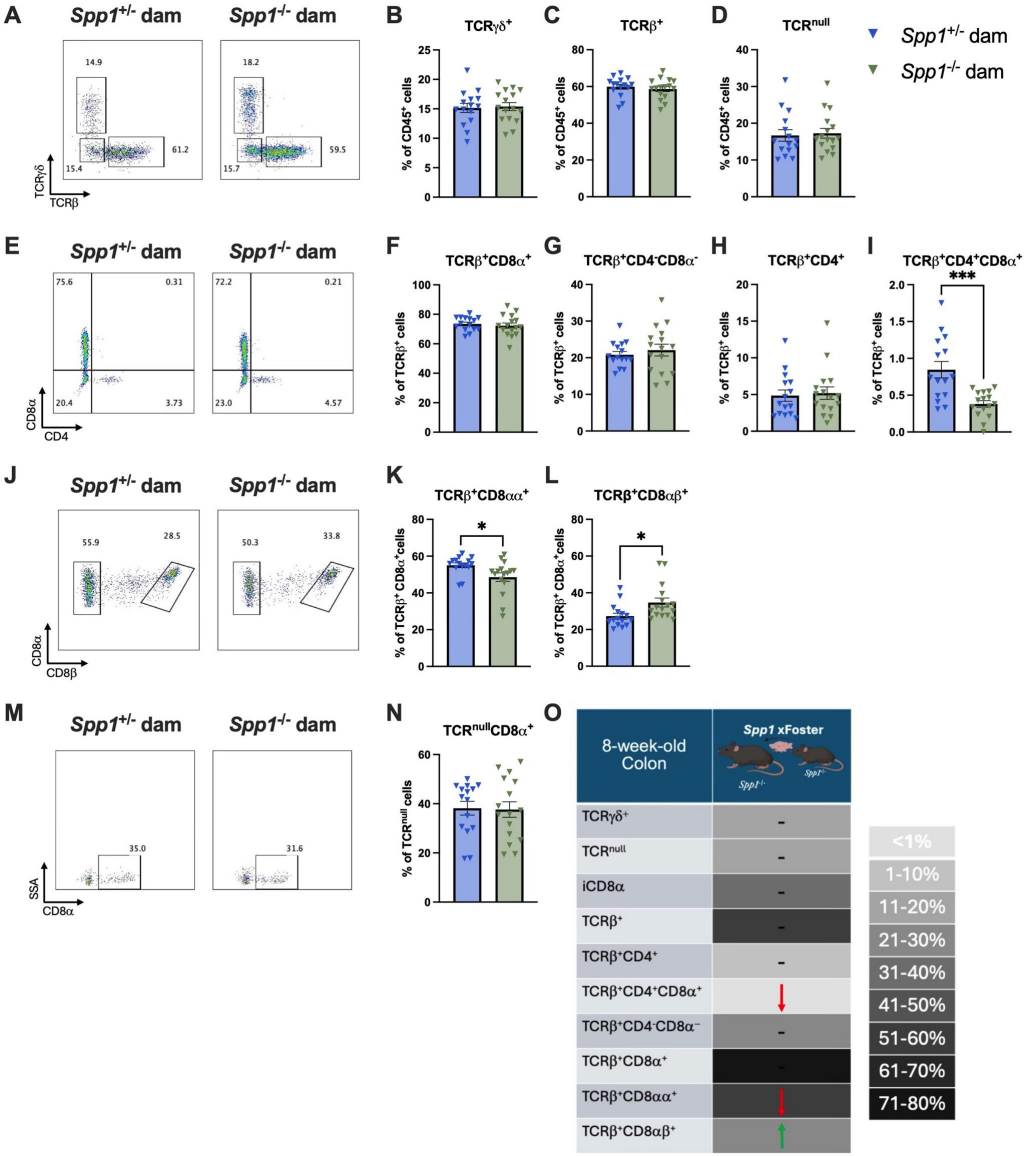
IEL frequencies in the colon of 8-week-old cross-fostered mice. (A) Representative flow plot of live CD45^+^ population gated by expression of TCR-β *x*-axis) and TCR-γ (*y*-axis). (B) Quantification of TCR-γ^+^ cells. (C) Quantification of TCR-β^+^ cells. (D) Quantification of TCR^null^ cells. (E) Representative flow plot of CD45^+^TCR-β^+^ population gated by expression of CD4 (*x*-axis) and CD8α (*y*-axis). (F) Quantification of TCR-β^+^CD8-α^+^ cells. (G) Quantification of TCR-β^+^CD4^-^CD8-α^−^ cells. (H) Quantification of TCRβ^+^CD4^+^ cells. (I) Quantification of TCR-β^+^CD4^+^CD8-α^+^ cells. (J) Representative flow plot of CD45^+^TCR-β^+^CD8-α^+^ population gated by expression of CD8-β *x*-axis). (K) Quantification of TCR-β^+^CD8-αα^+^ cells. (L) Quantification of TCR-β^+^CD8-αβ^+^ cells. (M) Representative flow plot of CD45^+^TCR^null^ population gated by expression of CD8-α *x*-axis). (N) Quantification of TCR^null^CD8α^+^ cells. (O) Summary of changes in 3-k-old small intestinal IEL. The prevalence of given IEL populations in mice raised by osteopontin-competent (*Spp1*^+/−^) dams is depicted by shading of the indicated cell (darker color equivalent to higher prevalence, key shown to right of table). Arrows within cells indicate an increase or decrease in prevalence of the given IEL population in pups raised by osteopontin-deficient (*Spp1*^−/−^) dams as compared to pups from osteopontin-competent (*Spp1*^+/−^) dams. Dashes indicate no significant difference in the given population. Each dot represents an individual mouse; *n* = 15 (*Spp1*^+/−^ dam), *n* = 16 (*Spp1*^−/−^ dam). Bars indicate SEM. Data are from2 independent experiments. *P ≤ 0.05, ***P ≤ 0.001.

These changes were less striking in the colon (Fig. 9A–N), though several patterns were conserved between organs, most notably the decrease in TCR-β^+^CD4^+^CD8-α^+^ IEL (Fig. 9I) and the shift in CD8-αα^+^/CD8-αβ^+^ IEL toward increased CD8αβ^+^ cells (Fig. 9J–L). No other differences were observed (Fig. 9A–H, M, N). A summary of these results in presented in Fig. 9O.

**Figure 10.**
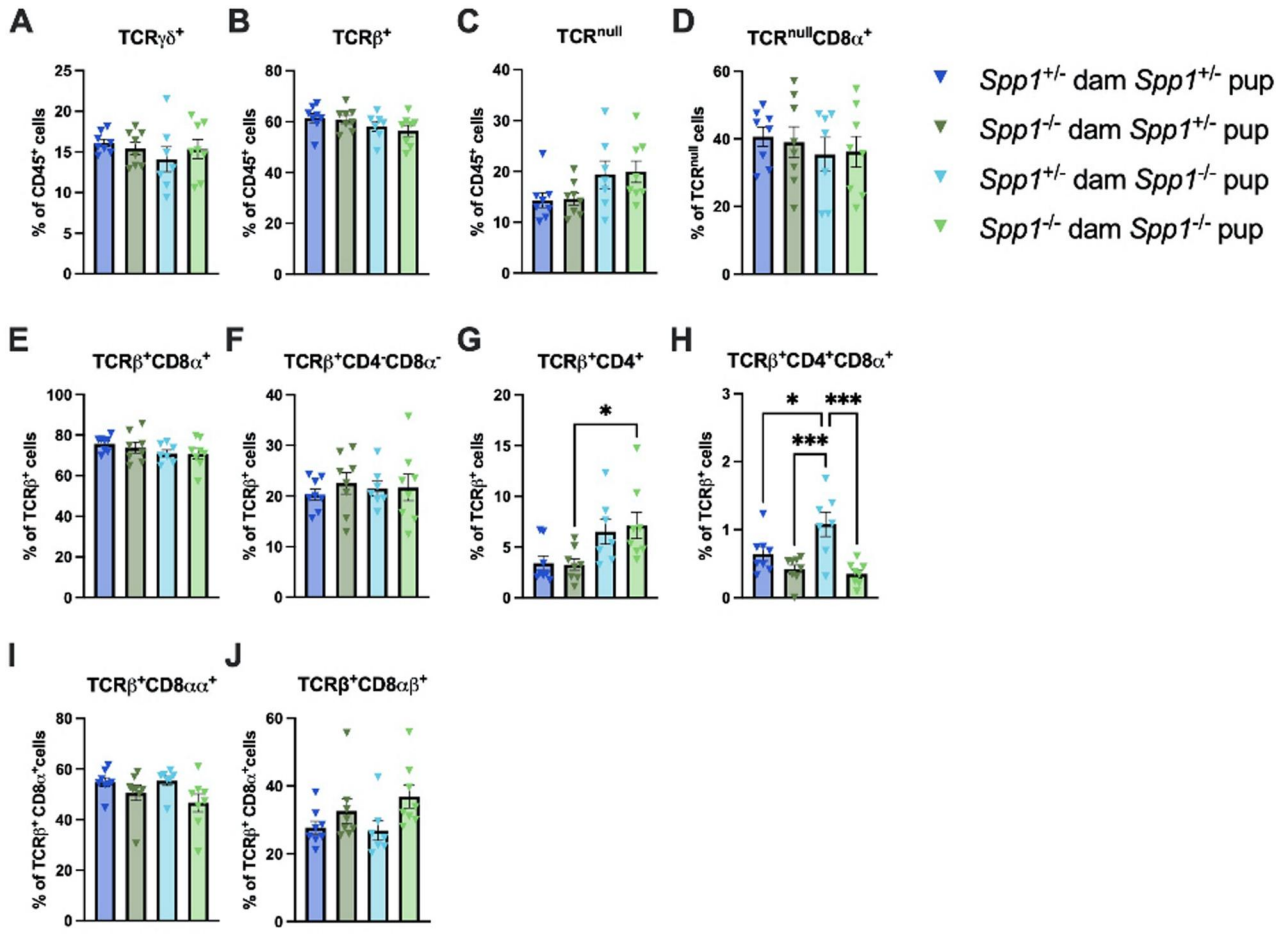
IEL frequencies and cell number in the colon of 8-week-old cross-fostered *Spp1*^+/−^ and *Spp1*^−/−^ mice. (A) Quantification of TCR-γ^+^ cells. (B) Quantification of TCR-β^+^ cells. (C) Quantification of TCR^null^ cells. (D) Quantification of TCR-β^+^CD8-α^+^ cells. (E) Quantification of TCR-β^+^CD4^-^CD8-α^−^ cells. (F) Quantification of TCR-β^+^CD4^+^ cells. (G) Quantification of TCR-β^+^CD4^+^CD8-α^+^ cells. (H) Quantification of TCR-β^+^CD8-αα^+^ cells. (I) Quantification of TCR-β^+^CD8-αβ^+^ cells. (J) Quantification of TCR^null^CD8-α^+^ cells. Each dot represents an individual mouse; *n* = 8 (*Spp1*^+/−^ dam *Spp1*^+/−^ pup), *n* = 8 (*Spp1*^−/−^ dam *Spp1*^+/−^ pup), *n* = 7 (*Spp1*^+/−^ dam *Spp1*^−/−^ pup), *n *= 8 (*Spp1*^−/−^ dam *Spp1*^−/−^ pup). Bars indicate SEM. Data are from 2 independent experiments. *P ≤ 0.05, ***P ≤ 0.001.

This pattern was also observed when comparing between all pups and dams (Fig. 10A–J). *Spp1*^−/−^ pups from *Spp1*^−/−^ foster dams displayed an increased frequency of TCR-β^+^CD4^+^ IEL as compared to *Spp1*^+/−^ pups from *Spp1*^−/−^ dams (Fig. 10G), and *Spp1*^−/−^ from *Spp1*^+/−^ dams displayed a higher percentage of TCR-β^+^CD4^+^CD8-α^+^ IEL as compared to all other groups (Fig. 10H); however, no differences were observed in other populations (Fig. 10A–F, I, J).

In summary, a number of changes were observed between the small intestinal and colonic IEL, though greater changes were observed in the small intestine (Figs. 7O, 9O), and few changes were observed when considering both dam and pup genotype.

We reasoned that the observed differences in IEL frequencies in mice fostered by osteopontin-competent and -deficient dams may be a reflection of increased proliferation and/or cell death. However, Ki67 and annexin V analysis showed that the differences in IEL frequencies may not be associated with varied proliferation and cellular death between the two groups (Supplemental Figures 2 and 3).

**Figure 11.**
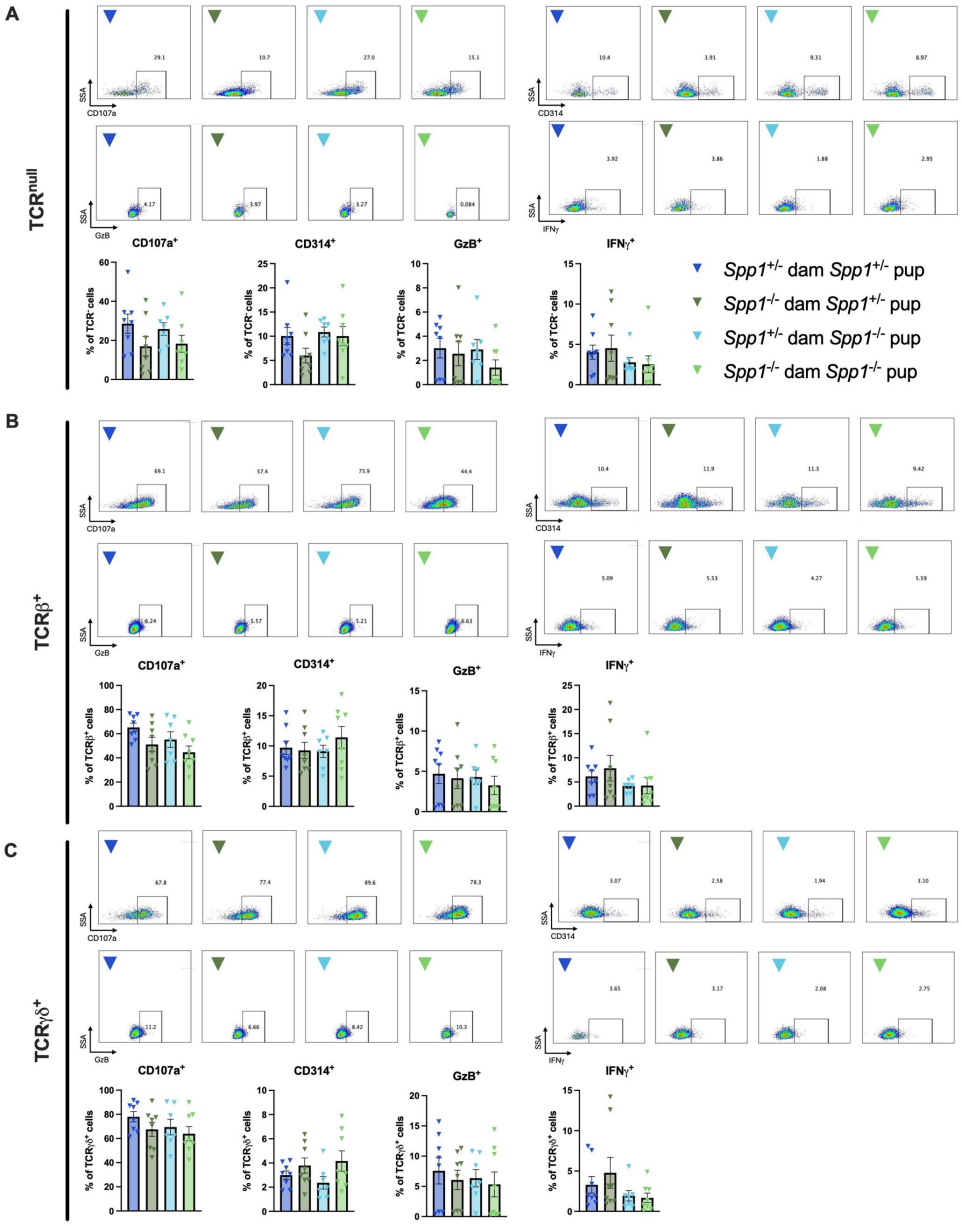
Cytotoxic and activation marker expression in small intestinal IEL derived from 8-wk-old *Spp1*^+/−^ and *Spp1*^−/−^ mice fostered by *Spp1*^+/−^ and *Spp1*^−/−^ dams. Percentage of cells in (A) TCR^null^ population; (B) TCR-β^+^ population; (C) TCR-γ^+^ population staining positive for the indicated markers. Each dot represents an individual mouse; *n* = 8 (*Spp1*^+/−^ dam *Spp1*^+/−^ pup), *n* = 8 (*Spp1*^−/−^ dam *Spp1*^+/−^ pup), *n* = 7 (*Spp1*^+/−^ dam *Spp1*^−/−^ pup), *n* = 8 (*Spp1*^−/−^ dam *Spp1*^−/−^ pup). Bars indicate SEM. Data are from three independent experiments. *P ≤ 0.05.

**Figure 12.**
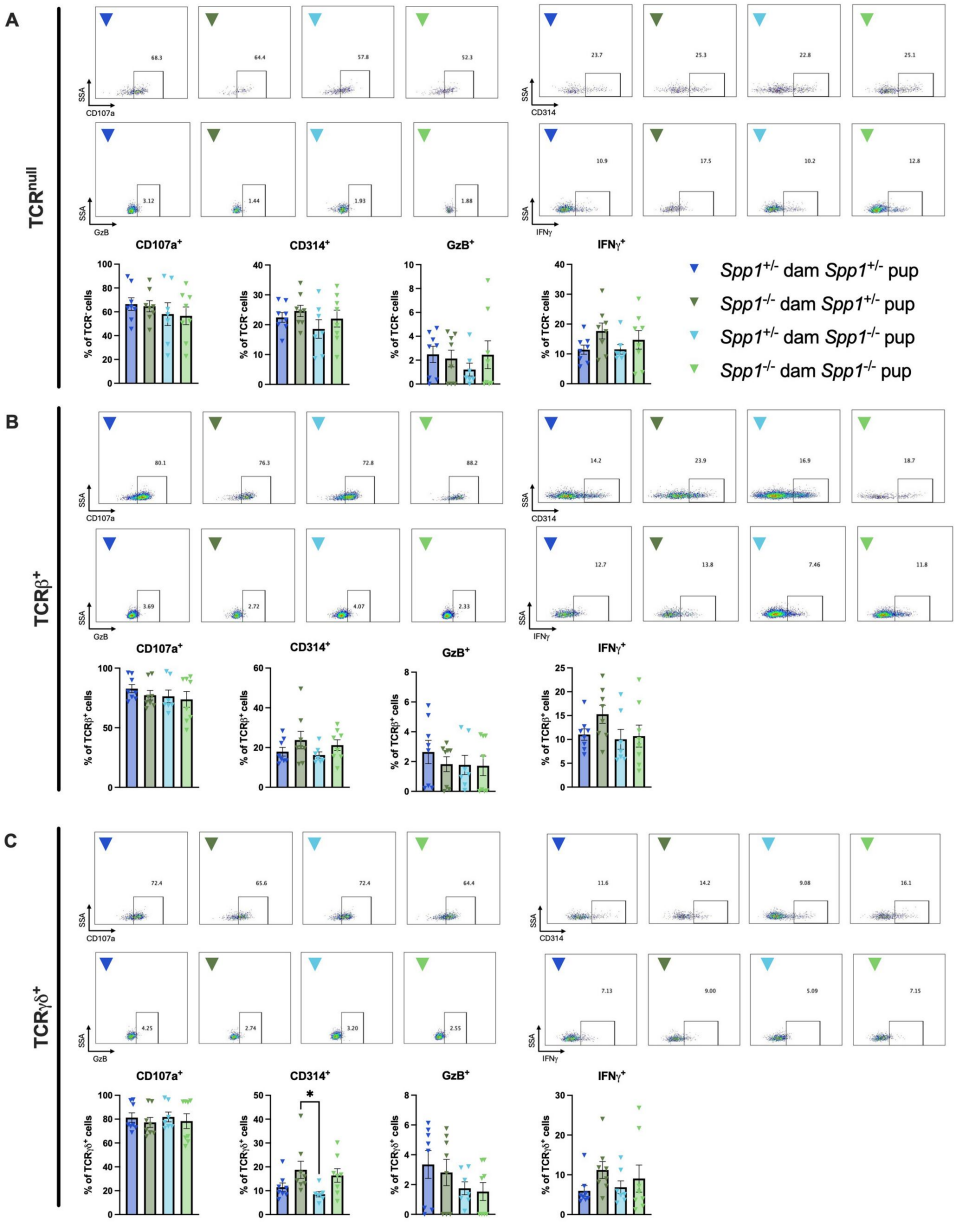
Cytotoxic and activation marker expression in colonic IEL derived from 8-wk-old *Spp1*^+/−^ and *Spp1*^-/-^ mice fostered by *Spp1*^+/−^ and *Spp1*^−/−^ dams. Percentage of cells in (A) TCR^null^ population; (B) TCR-β^+^ population; C. TCR-γ^+^ population staining positive for the indicated markers. Each dot represents an individual mouse; *n* = 8 (*Spp1*^+/−^ dam *Spp1*^+/−^ pup), *n* = 8 (*Spp1*^−/−^ dam *Spp1*^+/−^ pup), *n* = 7 (*Spp1*^+/−^ dam *Spp1*^−/−^ pup), *n* = 8 (*Spp1*^−/−^ dam *Spp1*^−/−^ pup). Bars indicate SEM. Data are from 3 independent experiments. *P ≤ 0.05.

The diversity of IEL populations is accompanied with varied effector functions. However, because many IEL express granzymes,^33^^,^^34^ it is believed that a great proportion of these cells are cytotoxic. To determine whether this particular effector function varied between IEL from 8w old mice fostered by *Spp1*^+/−^ and *Spp1*^−/−^ dams, we analyzed markers associated with cytotoxicity. However, no differences in intracellular granzyme B, IFN-γ or CD107a (a surrogate marker for degranulation) were observed in small intestinal or colonic TCR^null^, TCRβ^+^, or TCR-γ^+^ IEL (Figs 11A–C, 12A–C). The only observed difference was an increase in CD314 (a marker for cellular activation) in colonic TCR-γ^+^ IEL derived from *Spp1*^+/−^ pups from *Spp1*^−/−^ dams as compared to *Spp1*^−/−^ pups from *Spp1*^+/−^ dams (Fig. 12C).

**Figure 13.**
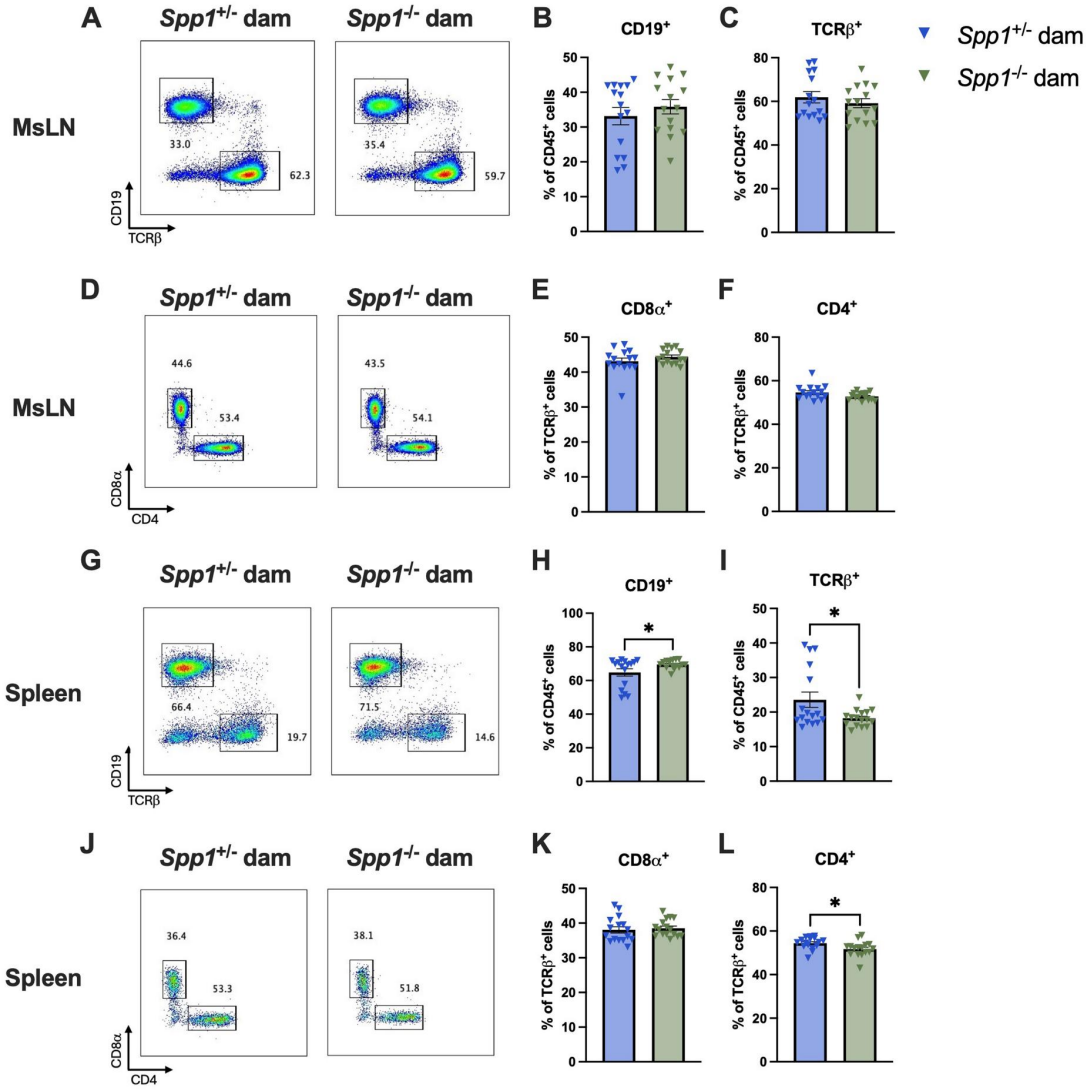
Lymphocyte frequencies in the mesenteric lymph nodes and spleen of 8-week-old cross-fostered mice. (A) Representative flow plot of MsLN live CD45^+^ population gated by expression of TCR-β *x*-axis) and CD19 (*y*-axis). (B) Quantification of CD19^+^ cells. (C) Quantification of TCR-β^+^ cells. (D) Representative flow plot of MsLN live CD45^+^TCR-β^+^ population gated by expression of CD4 *x*-axis) and CD8α (y-axis). (E) Quantification of CD8-α^+^ cells. (F) Quantification of CD4^+^ cells. (G) Representative flow plot of splenic live CD45^+^ population gated by expression of TCRβ *x*-axis) and CD19 (*y*-axis). (H) Quantification of CD19^+^ cells. (I) Quantification of TCRβ^+^ cells. (J) Representative flow plot of splenic live CD45^+^TCR-β population gated by expression of CD4 *x*-axis) and CD8-α (*y*-axis). (K) Quantification of CD8-α^+^ cells. (L) Quantification of CD4^+^ cells. Each dot represents an individual mouse; *n *= 15 (spleen), *n* = 16 (MsLN). Bars indicate SEM. Data are from 3 independent experiments. *P ≤ 0.05.

**Figure 14.**
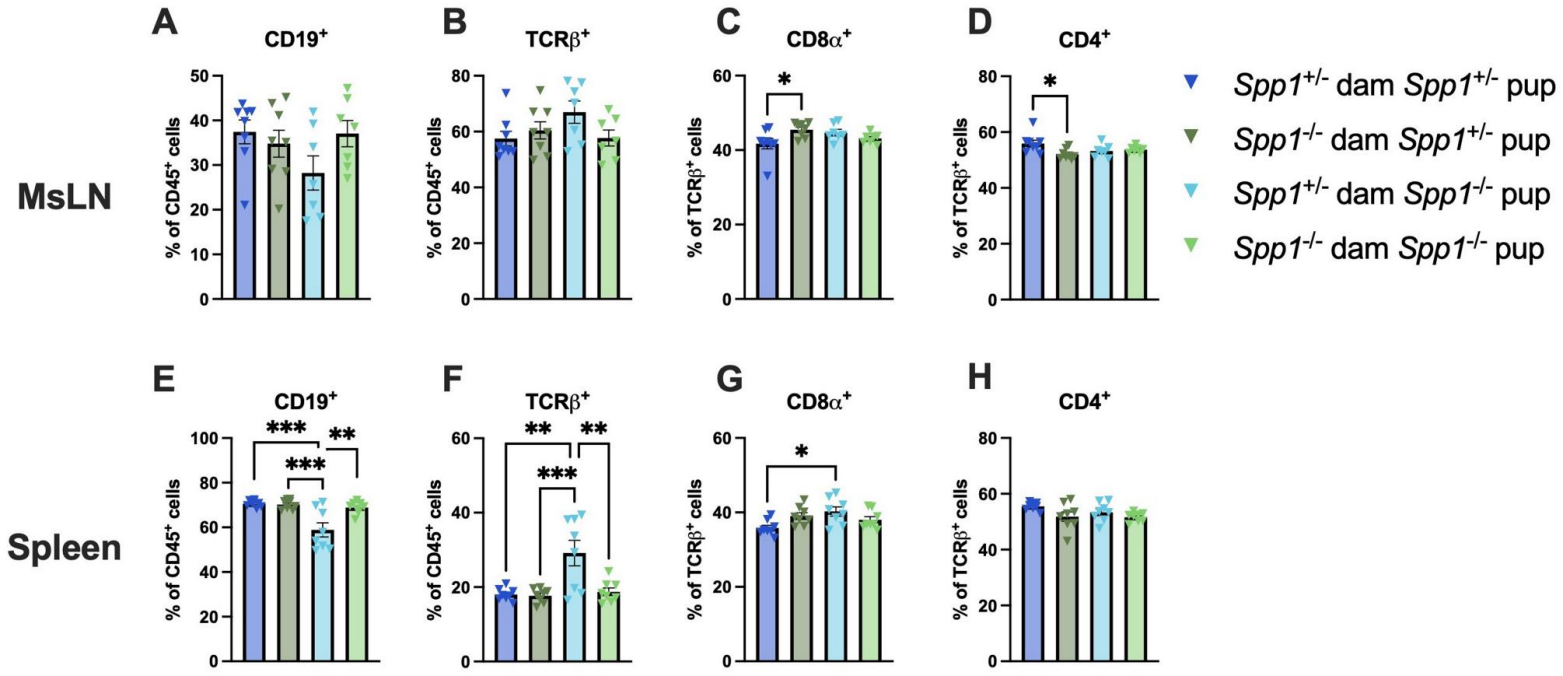
Lymphocyte frequencies and cell number in the mesenteric lymph nodes and spleen of 8-wk-old cross-fostered *Spp1*^+/−^ and *Spp1*^−/−^ mice. (A) Quantification of MsLN CD19^+^ cells. (B) Quantification of MsLN TCR-β^+^ cells. (C) Quantification of MsLN CD8-α^+^ cells. (D) Quantification of MsLN CD4^+^ cells. (E) Quantification of splenic CD19^+^ cells. (F) Quantification of splenic TCRp-β^+^ cells. (G) Quantification of splenic CD8-α^+^ cells. (H) Quantification of splenic CD4^+^ cells. Each dot represents an individual mouse; *n* = 8 (*Spp1*^+/−^ dam *Spp1*^+/−^ pup), *n* = 8 (*Spp1*^−/−^ dam *Spp1*^+/−^ pup), *n* = 7 (*Spp1*^+/−^ dam *Spp1*^−/−^ pup), *n* = 7 (*Spp1*^−/−^ dam *Spp1*^−/−^ pup). Bars indicate SEM. Data are from 3 independent experiments. *P ≤ 0.05, **P ≤ 0.01, ***P ≤ 0.001.

We also analyzed the effect of maternal osteopontin contribution in peripheral sites by analyzing cellular composition of both the spleen and MsLN. Gross cellularity of the MsLN was similar between the groups (Fig. 13A–F), while in the spleen, mice fostered by osteopontin-deficient dams presented with increased percentage of CD19^+^ cells and a decrease in TCR-β^+^ cells, specifically TCR-β^+^CD4^+^ cells (Fig. 13G–L). When taking into account pup and dam genotypes (Fig. 14A–P), these differences were minor and appear to be driven mainly by *Spp1*^−/−^ pups from *Spp1*^+/−^ dams (Fig. 14E, F). Similar to what was observed in the IEL compartment, no differences were observed in Annexin V or Ki67 staining of the spleen and MsLN (Fig. S4).

**Figure 15.**
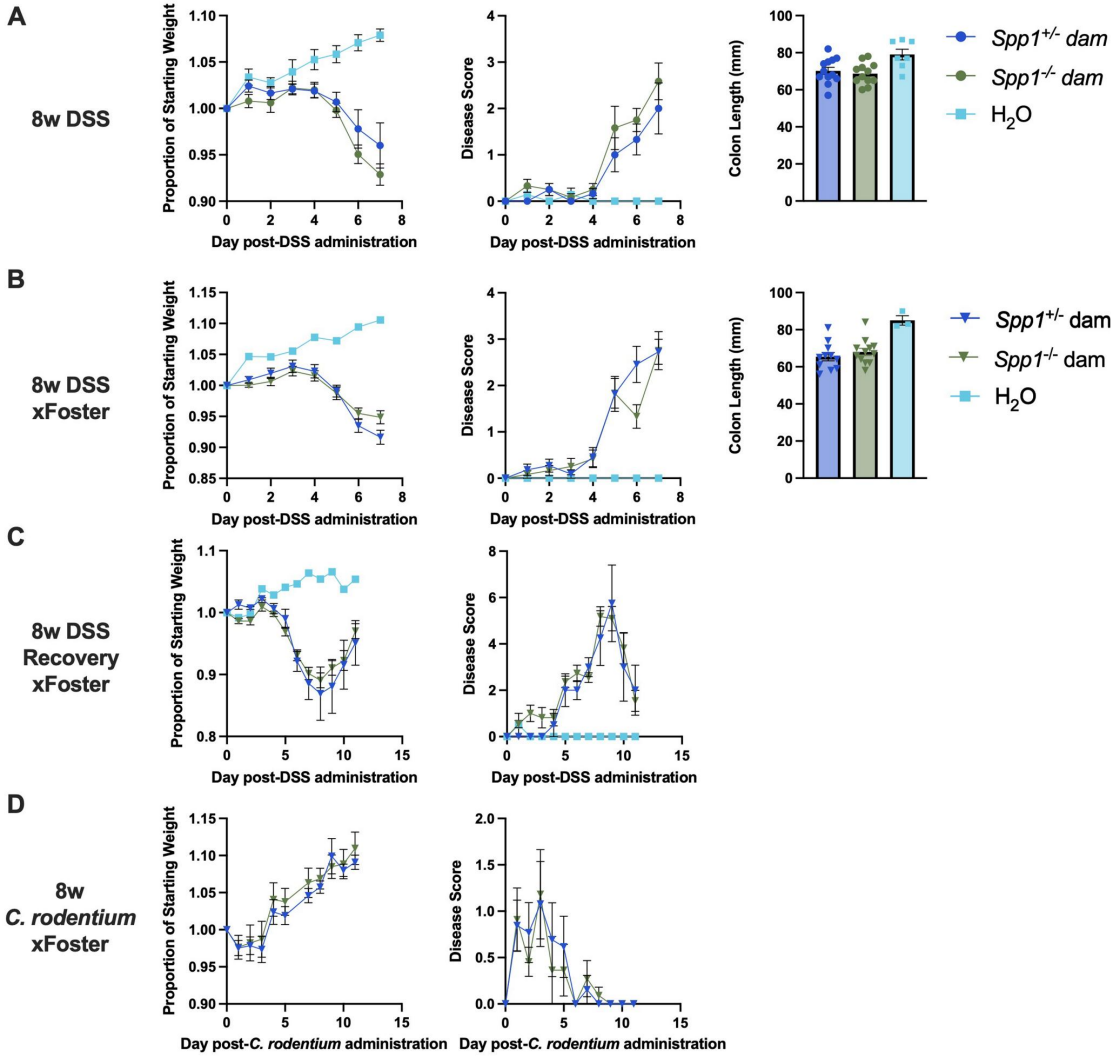
Response of 8-wk-old milk osteopontin-exposed and -non-exposed mice to models of intestinal inflammation. (A) Weight change (left), disease score (middle) and colon length (right) in the acute DSS model using *Spp1*^+/−^ mice birthed and reared by *Spp1*^+/−^ and *Spp1*^−/−^ dams; two independent experiments, *n* = 12 (*Spp1*^+/−^ dam), *n* = 12 (*Spp1*^−/−^ dam), *n* = 7 (H_2_O). (B) Weight change (left), disease score (middle) and colon length (right) in the acute DSS model using cross-fostered mice; one independent experiment, *n* = 11 (*Spp1*^+/−^ dam), *n* = 12 (*Spp1*^−/−^ dam), *n* = 3 (H_2_O) (C) Weight change (left) and disease score in the recovery DSS model with cross-fostered mice; three independent experiments, *n* = 4 (*Spp1*^+/−^ dam), *n* = 11 (*Spp1*^−/−^ dam), *n* = 2 (H_2_O). (D) *Citrobacter rodentium* infection with cross-fostered mice; 1 independent experiment, *n* = 13 (*Spp1*^+/−^ dam), *n* = 1 (*Spp1*^−/−^ dam).

Due to relevant IEL function in intestinal homeostasis and inflammation,^35–37^ we investigated whether the observed changes in the IEL compartment in the intestines of mice fostered by osteopontin-deficient dams resulted in increased susceptibility to intestinal inflammation. We utilized several models of intestinal inflammation, including DSS-induced colitis (acute inflammation and recovery) and *Citrobacter rodentium* infection. No differences between 8w mice from osteopontin-sufficient or -deficient mice were observed (Fig. 15).

## Discussion

In this report we show that the impact of milk osteopontin on developing IEL is complex and multifaceted. Nevertheless, some conserved patterns were observed. In both mouse models, a shift in the proportion of small intestinal TCR-β^+^CD8-αα^+^/TCRβ^+^CD8-αβ^+^ IEL was observed, with mice from osteopontin-deficient dams displaying an increased proportion of TCR-β^+^CD8-αβ^+^ IEL. Mice from these dams also displayed a decreased proportion of TCR-γ^+^ IEL in the small intestine. As >80% of TCR-γ^+^ IEL express CD8-αα, this may indicate an effect of milk osteopontin on cells expressing CD8-αα. Adding further evidence to this theory is the observed effect on TCR^null^CD8-α^+^, or iCD8-α, cells. Alterations in this subset were seen in both mouse models in the small intestine and colon.

Taken together, these data indicate that milk osteopontin exposure has an effect on the development or stability of CD8-αα^+^ IEL. The mechanism of this effect is beyond the scope of this publication and will require detailed and rigorous studies.

It is possible that some of the observed effects derive from differences in the microbiota between mice raised by *Spp1*^+/−^ and *Spp1*^−/−^ dams. IEL reside close to the intestinal lumen and are known to shape and be shaped by the microbiota.^38–40^  *Spp1*^−/−^ mice have been shown to have an altered microbiota from wild-type mice,^41^ and it is possible that maternal osteopontin acts on the developing microbiota rather than on IEL themselves, indirectly leading to the observed phenotypes. While we attempt to control for microbiota composition in these studies (using littermate dams that were derived from *Spp1*^+/−^ dams and only using pups from *Spp1*^+/−^ dams in the cross-foster experiments), it is likely that microbiota differences still exist between the groups. This will be an interesting avenue for future study and may help in elucidating the mechanism by which maternal osteopontin impacts CD8-αα^+^ IEL.

Although clear effects on the developing IEL compartment were observed in this study, the long-term effects of these changes are unclear. Despite differences in IEL composition, mice raised by osteopontin-deficient dams did not display increased susceptibility to DSS-induced colitis or to infection by *Citrobacter rodentium* at 8w of age. As the effects of early life osteopontin exposure appear to strongly affect cells expressing CD8-αα, it is possible that a model more dependent on these cells would be a better option to further probe the effects of maternal osteopontin exposure. However, CD8-αα^+^ IEL remain poorly understood, and their contributions to many intestinal diseases are unknown. It is also possible that the effects of maternal osteopontin exposure diminish over time, especially in osteopontin-competent mice. Exposing mice to models of inflammation and infection earlier in life (before 3 wk of age) may reveal more drastic phenotypes due to differences in the total amount of osteopontin present in the intestines at these times.

Differences between endogenous intestinal osteopontin and milk-derived osteopontin are poorly understood. We describe an effect based on maternal osteopontin exposure even when controlling for pup genotype (Figs 7–10), indicating that maternal osteopontin has a distinct function from the endogenous osteopontin found in the juvenile intestine. Interestingly, the effect of maternal genotype on IEL development was much stronger than that of pup genotype, providing further evidence for differential action of the isoforms. This may be partly due to an increase in total osteopontin concentration but is likely also due to differences between the isoforms. Milk osteopontin undergoes extensive post-translational modification,^42^ which may result in differences in bioactivity compared to endogenous intestinal forms. Milk osteopontin also passes through the stomach before arriving in the intestine, resulting in partial digestion and exposure of bioactive sites.^43^ In addition, milk-derived osteopontin has been shown to form a complex with lactoferrin, altering its bioactivity.^44^ The observed effects of milk-derived osteopontin likely result from the summation of several structural and functional differences.

It is important to make a cautionary point. The number of pups reared by each dam were not standardized among the 2 different models used in this report. Variations on litter size may have unforeseen impact on the quality of milk components, including osteopontin, which in turn may affect the composition of the IEL compartment. However, we have not observed significant variability in size litter between *Spp1*^+/−^ and *Spp1*^−/−^ dams. In addition, all our dams were littermates and of similar age. Thus, potential variation in milk quality is probably homogenized in the dam cohorts, indicating that what the differences presented in this manuscript are likely due to the presence or absence of osteopontin.

The authors find it important to note that the *Spp1* cross-foster mice were not the only mice generated for this study. We began by generating a MMTV-Cre^+^  *Spp1*^fl/fl^ mouse by backcrossing mixed-background MMTV-Cre^+/−^ mice (Tg(MMTV-cre)4Mam/J, Jackson Labs no. 003553) onto our previously described *Spp1*^fl/fl^ mice.^30^ These mice carried Cre recombinase under the control of the Mouse Mammary Tumor Virus Long Terminal Repeat promoter, which should have allowed for excision of the floxed allele in the mammary gland. However, these mice excised a floxed allele multiple times throughout their lineage, even in mice lacking expression of Cre recombinase. After intense screening and careful management, we were unable to ever recover these mice back to their *Spp1*^fl/fl^ background, which remains an unexplained genetic phenomenon. We have never seen this phenomenon in *Spp1*^fl/fl^ mice carrying Cre recombinase under any other promoter. The authors offer this as a note of caution to anyone attempting to generate these mice and suggest the use of a more tractable Cre line, such as the whey acidic protein Cre (WAP-Cre).

In summary, this report offers a preliminary description of the impact of milk-derived osteopontin on the structure of the intestinal IEL compartment. Although the effects are varied, there is evidence that the most affected populations are those expressing CD8-αα. Further studies will be needed to investigate the mechanisms by which milk-derived osteopontin exerts its effect on IELs early in life, and the potential modulation of IEL effector functions.

## Supplementary Material

vlaf057_Supplementary_Data

## Data Availability

The authors confirm the data supporting the finding of this study are available within the article and its supplementary materials.
