# Supplementary material for: Milk-derived osteopontin influences the composition of the intestinal intraepithelial lymphocyte compartment
Source: Immunohorizons. 2025 Oct 9;9(11):vlaf057. doi: 10.1093/immhor/vlaf057 (PMC12597887; doi:10.1093/immhor/vlaf057)
Supplement: vlaf057_Supplementary_Data [file vlaf057_supplementary_data.pdf]

Supplementary Figure Legends

Figure S1: Gating strategies for flow cytometry experiments. **A.** Gating of IEL populations in small intestine and colon. **B.** Gating of lymphocyte populations in spleen and MsLN.

Figure S2: Ki67 intracellular staining and Annexin V extracellular staining of small intestinal IEL populations derived from 8-week-old *Spp1*$^{+/-}$ and *Spp1*$^{-/-}$ mice fostered by *Spp1*$^{+/-}$ and *Spp1*$^{-/-}$ dams. Plots show percentage of cells in each IEL population staining positive for Ki67 or Annexin V. Each dot represents an individual mouse. **A.** TCR^{null} IEL; **B.** TCRβ^{+} IEL; **C.** TCRγδ^{+} IEL. Annexin V: n=8 (*Spp1*$^{+/-}$ dam *Spp1*$^{+/-}$ pup), n=8 (*Spp1*$^{-/-}$ dam *Spp1*$^{+/-}$ pup), n=6 (*Spp1*$^{+/-}$ dam *Spp1*$^{-/-}$ pup), n=7 (*Spp1*$^{-/-}$ dam *Spp1*$^{-/-}$ pup); Ki67: n=4 (*Spp1*$^{+/-}$ dam *Spp1*$^{+/-}$ pup), n=4 (*Spp1*$^{-/-}$ dam *Spp1*$^{+/-}$ pup), n=5 (*Spp1*$^{+/-}$ dam *Spp1*$^{-/-}$ pup), n=6 (*Spp1*$^{-/-}$ dam *Spp1*$^{-/-}$ pup). Bars indicate SEM. Data are from three independent experiments.

Figure S3: Ki67 intracellular staining and Annexin V extracellular staining of colonic IEL populations derived from 8-week-old *Spp1*$^{+/-}$ and *Spp1*$^{-/-}$ mice fostered by *Spp1*$^{+/-}$ and *Spp1*$^{-/-}$ dams. Plots show percentage of cells in each IEL population staining positive for Ki67 or Annexin V. Each dot represents an individual mouse. **A.** TCR^{null} IEL; **B.** TCRβ^{+} IEL; **C.** TCRγδ^{+} IEL. Annexin V: n=8 (*Spp1*$^{+/-}$ dam *Spp1*$^{+/-}$ pup), n=7 (*Spp1*$^{-/-}$ dam *Spp1*$^{+/-}$ pup), n=8 (*Spp1*$^{+/-}$ dam *Spp1*$^{-/-}$ pup), n=7 (*Spp1*$^{-/-}$ dam *Spp1*$^{-/-}$ pup); Ki67: n=4 (*Spp1*$^{+/-}$ dam *Spp1*$^{+/-}$ pup), n=4 (*Spp1*$^{-/-}$ dam *Spp1*$^{+/-}$ pup), n=5 (*Spp1*$^{+/-}$ dam *Spp1*$^{-/-}$ pup), n=6 (*Spp1*$^{-/-}$ dam *Spp1*$^{-/-}$ pup). Bars indicate SEM. Data are from three independent experiments.

Figure S4: Ki67 and Annexin V staining of **A.** Mesenteric lymph node and **B.** Splenic lymphocytes from 8-week-old *Spp1*$^{+/-}$ and *Spp1*$^{-/-}$ mice fostered by *Spp1*$^{+/-}$ and *Spp1*$^{-/-}$ dams. Plots show percentage of cells in each population staining positive for Ki67 or Annexin V. Each dot represents an individual mouse. MsLN Ki67: n=7 (*Spp1*$^{+/-}$ dam *Spp1*$^{+/-}$ pup), n=8 (*Spp1*$^{-/-}$ dam *Spp1*$^{+/-}$ pup), n=7 (*Spp1*$^{+/-}$ dam *Spp1*$^{-/-}$ pup), n=8 (*Spp1*$^{-/-}$ dam *Spp1*$^{-/-}$ pup); MsLN Annexin V: n=7 (*Spp1*$^{+/-}$ dam *Spp1*$^{+/-}$ pup), n=8 (*Spp1*$^{-/-}$ dam *Spp1*$^{+/-}$ pup), n=6 (*Spp1*$^{+/-}$ dam *Spp1*$^{-/-}$ pup), n=8 (*Spp1*$^{-/-}$ dam *Spp1*$^{-/-}$ pup); Spleen Ki67**:** n=8 (*Spp1*$^{+/-}$ dam *Spp1*$^{+/-}$ pup), n=8 (*Spp1*$^{-/-}$ dam *Spp1*$^{+/-}$ pup), n=8 (*Spp1*$^{+/-}$ dam *Spp1*$^{-/-}$ pup), n=8 (*Spp1*$^{-/-}$ dam *Spp1*$^{-/-}$ pup); Spleen Annexin V**:** n=8 (*Spp1*$^{+/-}$ dam *Spp1*$^{+/-}$ pup), n=8 (*Spp1*$^{-/-}$ dam *Spp1*$^{+/-}$ pup), n=8 (*Spp1*$^{+/-}$ dam *Spp1*$^{-/-}$ pup), n=7 (*Spp1*$^{-/-}$ dam *Spp1*$^{-/-}$ pup). Bars indicate SEM. Data are from three independent experiments.

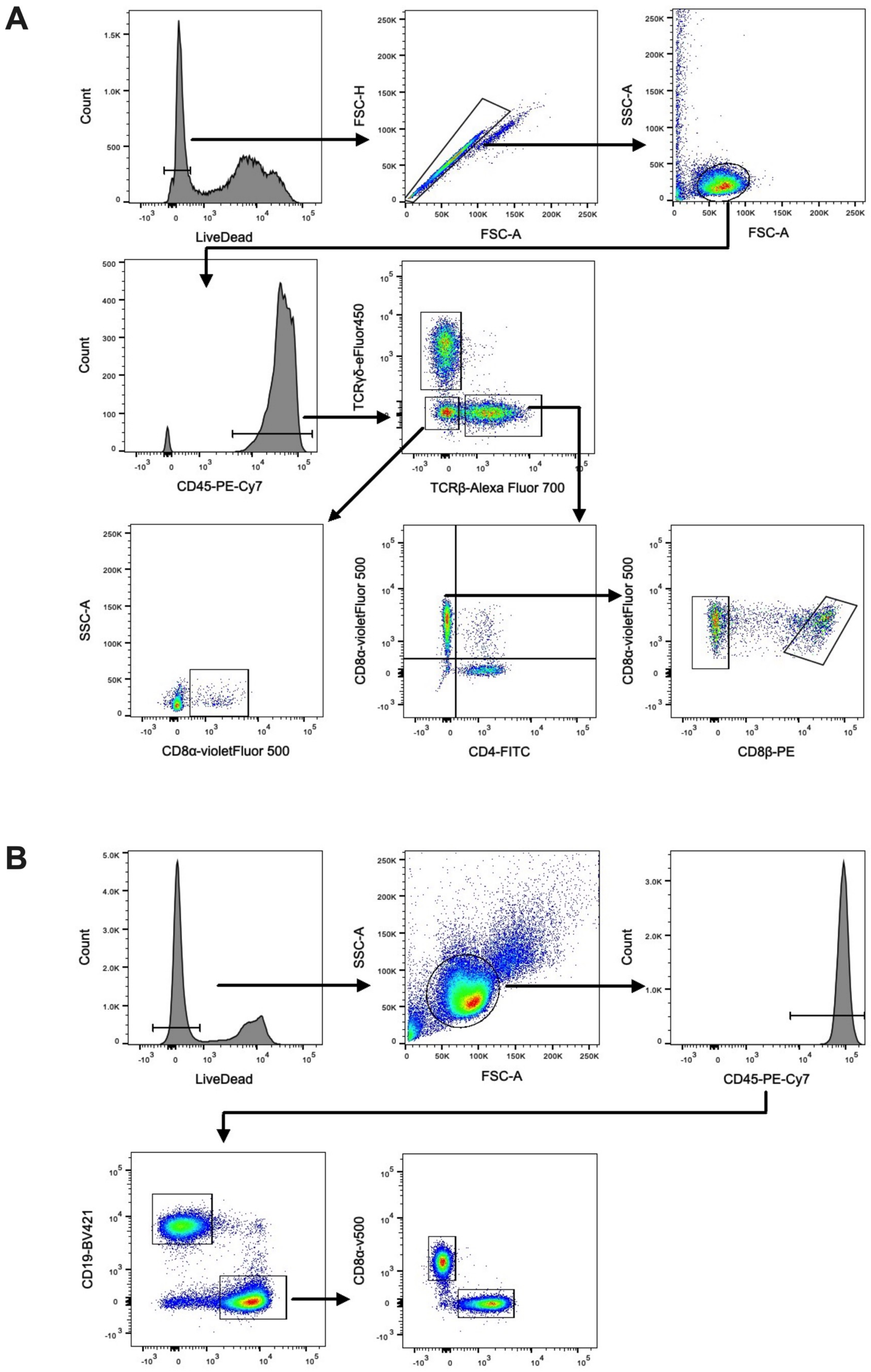

Supp. Figure 1

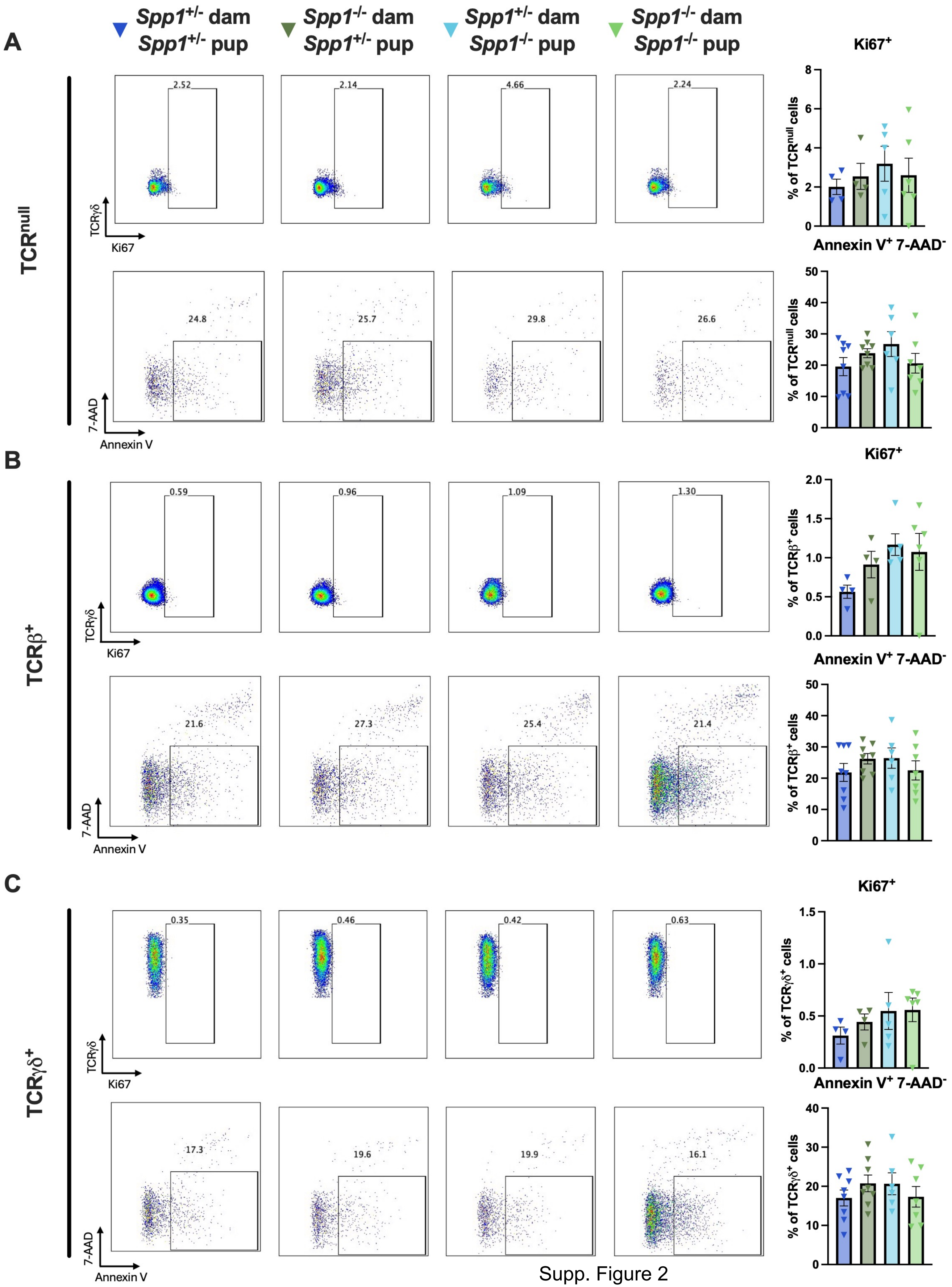

Supp. Figure 2

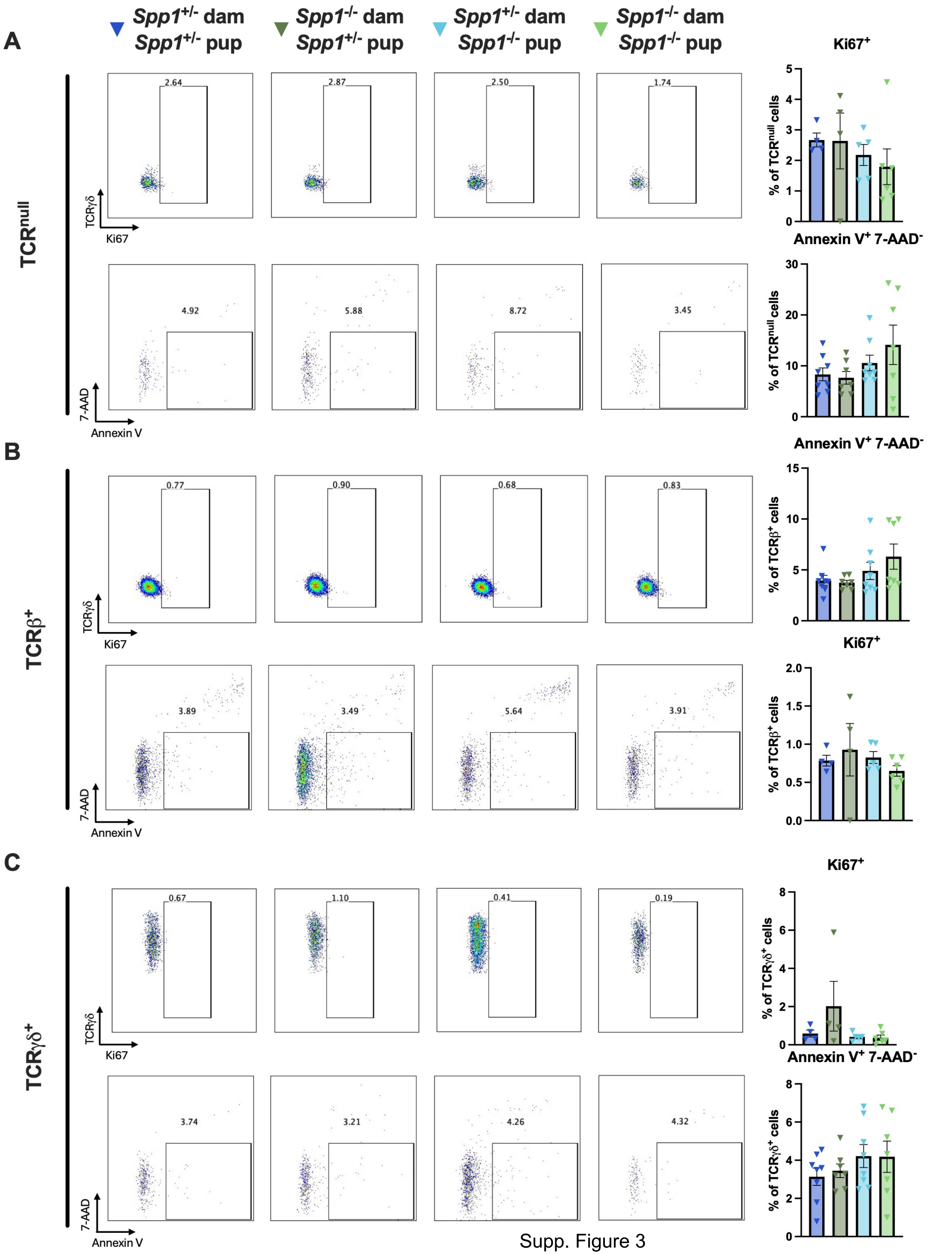

Supp. Figure 3

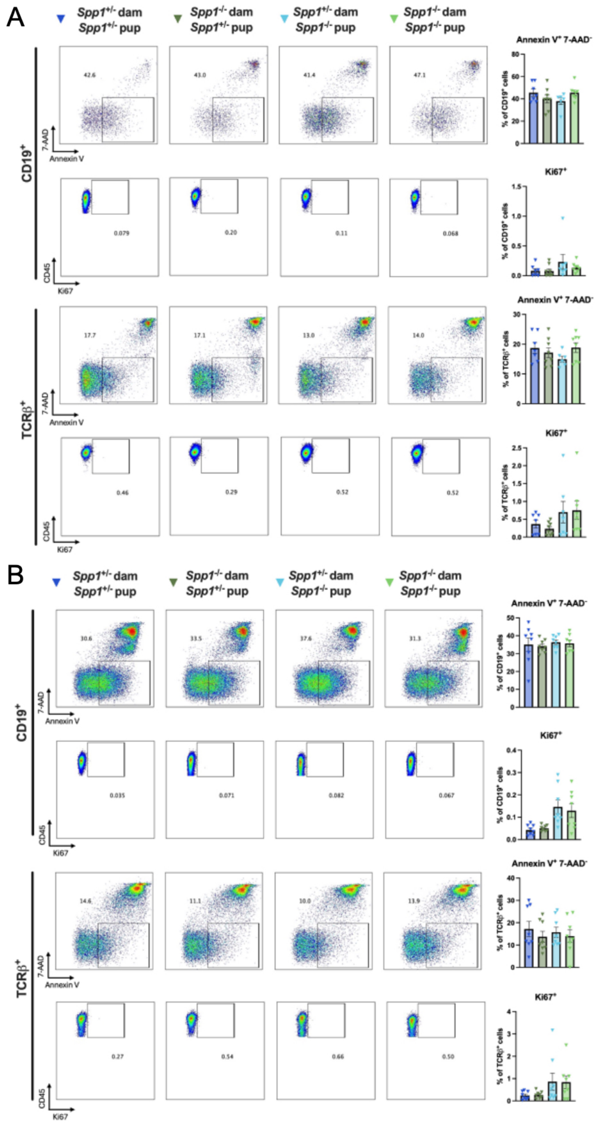

Supp. Figure 4
